# SIRT7-dependent deacetylation of the U3-55k protein controls pre-rRNA processing

**DOI:** 10.1038/ncomms10734

**Published:** 2016-02-12

**Authors:** Sifan Chen, Maximilian Felix Blank, Aishwarya Iyer, Bingding Huang, Lin Wang, Ingrid Grummt, Renate Voit

**Affiliations:** 1 Division of Molecular Biology of the Cell II, German Cancer Research Center, DKFZ-ZMBH Alliance, Im Neuenheimer Fed 581, 69120 Heidelberg, Germany; 2 Division of Theoretical Bioinformatics, German Cancer Research Center, 69120 Heidelberg, Germany; 3 Genomics and Proteomics Core Facility, German Cancer Research Center, 69120 Heidelberg, Germany

## Abstract

SIRT7 is an NAD^+^-dependent protein deacetylase with important roles in ribosome biogenesis and cell proliferation. Previous studies have established that SIRT7 is associated with RNA polymerase I, interacts with pre-ribosomal RNA (rRNA) and promotes rRNA synthesis. Here we show that SIRT7 is also associated with small nucleolar RNP (snoRNPs) that are involved in pre-rRNA processing and rRNA maturation. Knockdown of SIRT7 impairs U3 snoRNA dependent early cleavage steps that are necessary for generation of 18S rRNA. Mechanistically, SIRT7 deacetylates U3-55k, a core component of the U3 snoRNP complex, and reversible acetylation of U3-55k modulates the association of U3-55k with U3 snoRNA. Deacetylation by SIRT7 enhances U3-55k binding to U3 snoRNA, which is a prerequisite for pre-rRNA processing. Under stress conditions, SIRT7 is released from nucleoli, leading to hyperacetylation of U3-55k and attenuation of pre-rRNA processing. The results reveal a multifaceted role of SIRT7 in ribosome biogenesis, regulating both transcription and processing of rRNA.

## Abstract (web summary)

SIRT7 is a protein deacetylase with important roles in rRNA synthesis, ribosome biogenesis and cell proliferation. Here the authors show a role of SIRT7 in rRNA maturation via deacetylation of U3-55k, a core component of the U3 snoRNP complex.

## Graphical abstract

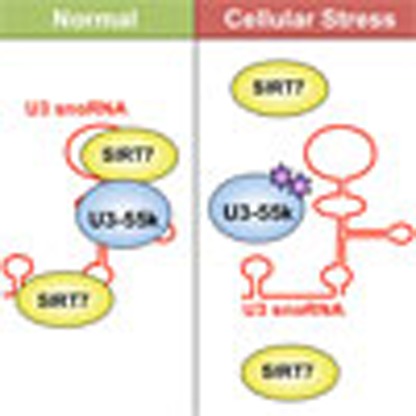

Ribosome biogenesis is a highly regulated process that requires the coordinated activity of all three nuclear DNA-dependent RNA polymerases (Pol I, II and III) along with more than 200 trans-acting factors, including transcription factors, small nucleolar RNPs (snoRNPs), ribosomal proteins, and proteins that promote processing and modification of ribosomal RNA (rRNA)^1^,^2^,^3^. The initial 47S ribosomal precursor RNA (pre-rRNA) is posttranscriptionally cleaved to form the mature 28S, 18S and 5.8S rRNAs. During the maturation process, the pre-rRNA and its processing intermediates undergo numerous posttranscriptional modifications, which are guided and catalysed by snoRNPs (ref. 4).

In eukaryotes, the U3 snoRNA-containing snoRNP is essential for processing of pre-rRNA (refs 4, 5). U3 snoRNA is associated with four common box C/D core snoRNP proteins, that is, 15.5k, Nop56, Nop58, and fibrillarin and the U3-specific protein U3-55k (refs 4, 6). The 12S U3 snoRNP particle constitutes a subcomplex of the phylogenetically conserved 80S/2.2 MDa small-subunit (SSU) processome, a large ribonucleoprotein complex that assembles on nascent pre-rRNA and is indispensable for ribosome biogenesis^7^,^8^,^9^,^10^. The yeast SSU processome contains as many as 72 proteins, including endonucleases, RNA helicases, ATPases, GTPases, protein kinases and other regulatory proteins^11^. The U3 snoRNA was implicated in pre-rRNA processing by chemical cross-linking and mutational studies, showing that regions of complementarity allow base pairing of U3 snoRNA with the 5′-ETS and pre-18S rRNA, thus directing pre-rRNA cleavage^12^,^13^,^14^,^15^,^16^. Conditional knockout of the *U3 snoRNA* genes in yeast abolished pre-rRNA processing at specific sites, leading to accumulation of unprocessed 35S pre-rRNA and loss of mature 18S rRNA (ref. 17).

For many years, research on mammalian pre-rRNA processing lagged behind that on budding yeast, mainly because of the power of yeast genetics. A recent screen in human cells identified 286 proteins involved in pre-rRNA synthesis and pre-rRNA maturation, 74 of them having no yeast homologue^2^. Among the identified genes was *SIRT7*, which encodes a member of the sirtuin family of NAD^+^-dependent deacetylases. Sirtuins constitute a phylogenetically conserved protein family with central roles in the cellular response to oxidative, metabolic and genotoxic stress^18^,^19^,^20^. Sirtuins are regarded as intracellular sensors of the metabolic environment, as the enzymatic activity is dependent on the cosubstrate NAD^+^ (refs 21, 22). Little is known about SIRT7 function and only a few molecular substrates of SIRT7 have been identified. SIRT7 is enriched in nucleoli, where it facilitates RNA polymerase I (Pol I)-dependent transcription by interacting with the upstream binding factor (UBF; ref. 23) and the Pol I subunit PAF53 (ref. 24). Interaction with and deacetylation of PAF53 by SIRT7 drives ribosomal DNA (rDNA) transcription and ribosome biogenesis, and consequently, cell growth and proliferation^24^,^25^. SIRT7 expression correlates with cell growth and proliferation, being abundant in metabolically active cells, and low or even absent in non-proliferating cells^25^. Consistent with a functional link between SIRT7, ribosome biogenesis and cell proliferation, SIRT7 is overexpressed in several types of tumours^26^,^27^,^28^.

In accord with previous studies showing that pre-rRNA transcription and processing are functionally coupled^8^,^29^,^30^, we report here that SIRT7 plays an essential role not only in rDNA transcription but also in specific cleavage of pre-rRNA. We show that SIRT7 is associated with U3 snoRNPs and promotes pre-rRNA cleavage at the 5′-terminal processing site by deacetylating U3-55k (Rrp9), a core subunit of the U3 snoRNP complex, deacetylation of U3-55k being required for all subsequent processing events. On exposure to hyperosmotic stress, SIRT7 is released from nucleoli, leading to hyperacetylation of U3-55k and processing defects. The results uncover a SIRT7-dependent mechanism that links rDNA transcription to pre-rRNA processing, reinforcing the pivotal role of SIRT7 in ribosome biogenesis, cell metabolism and homeostasis.

## Results

### SIRT7 is associated with snoRNAs

**Figure 1:**
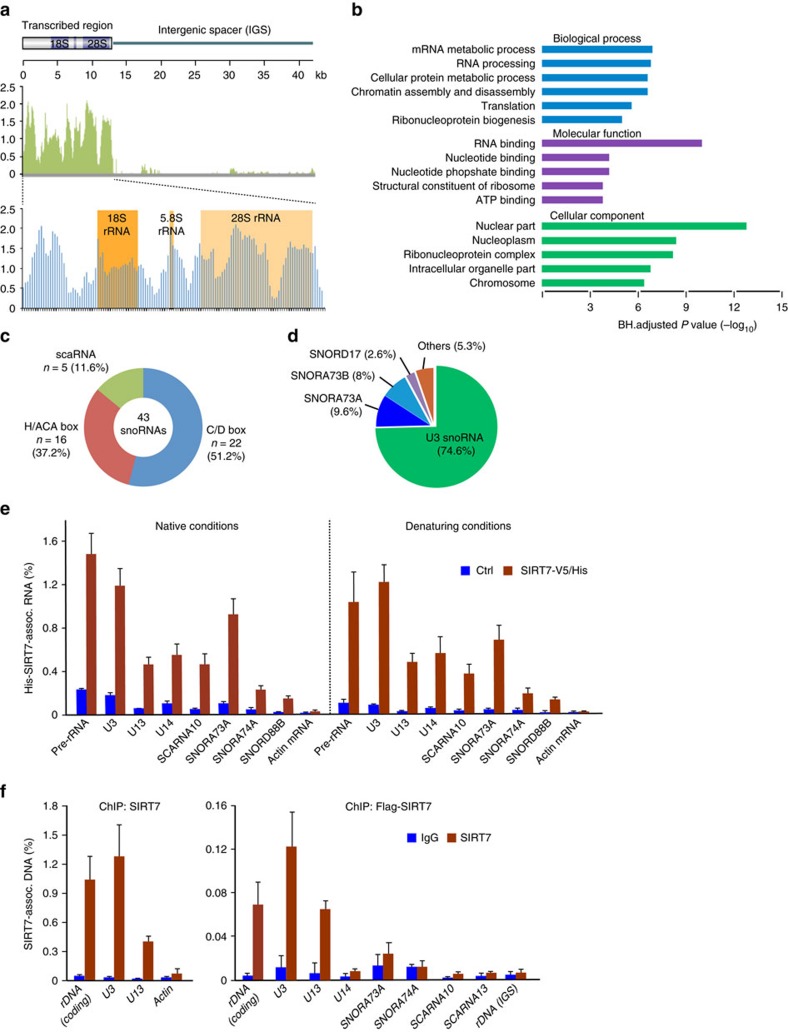
SIRT7 is associated with pre-rRNA and snoRNAs. (**a**) SIRT7 CLIP-seq reads mapped to a custom annotation file of a human rDNA repeat (middle) or the transcribed region (bottom). The region encoding 18S, 5.8S and 28S rRNA is highlighted. SIRT7 reads after subtraction of IgG reads were normalized to input reads (*y* axis). (**b**) Gene ontology categories of SIRT7 CLIP-seq peaks. The most representative clusters are shown according to the ajusted *P* value (−log_10_). (**c**) SIRT7-bound snoRNAs comprise C/D box, H/ACA box snoRNAs and scaRNAs. The number (*n*) and relative abundance (%) of each snoRNA class associated with SIRT7 is presented. (**d**) U3, SNORA73A and 73B snoRNAs are overrepresented among SIRT7-associated snoRNAs. SIRT7 reads mapped to corresponding snoRNAs are indicated as percentage of all snoRNAs identified by CLIP-seq. (**e**) Comparison of SIRT7-associated RNAs under native and denaturing conditions. His/V5-tagged SIRT7 expressed in HEK293T cells was affinity-purified on Ni-NTA-agarose under native or denaturing conditions, and associated RNAs were detected by RT–qPCR. Lysates from non-transfected HEK293T cells were used for control (Ctrl). Associated pre-RNA was monitored by RT–qPCR using primer H1 (Supplementary Table 3). Bars represent means±s.d. from three experiments. See also Supplementary Fig. 2b,d. (**f**) ChIP assays showing association of endogenous SIRT7 (left panel) or transiently overexpressed Flag-SIRT7 (right panel) with the indicated gene loci in HEK293T cells. rDNA was amplified using primers H4 (coding) and H18 (IGS; Supplementary Table 3). Bars represent means±s.d. from three experiments. See also Supplementary Fig. 2d.

To characterize the repertoire of nuclear RNAs that are associated with SIRT7, we performed cross-linking and immunoprecipitation (CLIP)-seq, that is, immunoprecipitated Flag-tagged SIRT7 from UV-cross-linked HEK293T cells and sequenced the co-precipitated RNA. Deep sequencing of SIRT7-associated RNAs and subsequent bioinformatic analysis identified pre-rRNA, numerous messenger RNAs (mRNAs) and non-coding RNAs (Supplementary Fig. 1a). Consistent with SIRT7 being associated with Pol I and activating rDNA transcription^24^, the majority of SIRT7-associated RNA reads (87.8%) covered the entire transcribed region of rDNA, supporting that SIRT7 binds to nascent pre-rRNA (Fig. 1a and Supplementary Fig. 1b,c). About 9.7% of reads mapped to transcripts synthesized by Pol II and 0.8% to RNAs synthesized by Pol III. Among the SIRT7-associated Pol II and Pol III transcripts, RNAs implicated in RNA metabolism were enriched, including mRNAs encoding ribosomal proteins and snoRNAs (Fig. 1b). We identified a total of 43 snoRNAs, comprising all three classes of snoRNA, that is, box C/D, box H/ACA and small Cajal body-specific (sca) RNAs (Fig. 1c and Supplementary Table 1). Two of them, U3 and U13 snoRNA, are transcribed from their own promoter, U3 RNA being the most abundant one (Fig. 1d and Supplementary Fig. 1d). In addition, numerous intron-encoded snoRNAs were associated with SIRT7, including SNORA73A, SNORA73B, SNORA74A, SCARNA6, SCARNA13 and SCARNA10, the distribution of reads at the coding region of these snoRNA being markedly higher than at adjacent genomic regions, indicating that SIRT7 is associated with mature snoRNAs (Supplementary Fig. 2a). To validate that SIRT7 binds to mature U3 snoRNA rather than the U3 snoRNA precursor (pre-U3 snoRNA), we performed reverse transcription–quantitative polymerase chain reaction (RT–qPCR) of U3 snoRNA associated with Flag-tagged SIRT7 using primers covering the 8 nt extension at the 3′ end of pre-U3 snoRNA. This analysis, together with alignment of the U3 snoRNA CLIP-seq reads, revealed that SIRT7 predominantly interacts with mature and not with pre-U3 snoRNA (Supplementary Fig. 1e,f).

To prove that the interaction of SIRT7 with snoRNAs is direct rather than mediated by auxiliary proteins, we repeated the CLIP experiments under native and denaturing conditions, under which only direct protein/RNA interactions are preserved. Analysis of recovered RNA by RT–qPCR confirmed direct binding of SIRT7 to pre-rRNA and to snoRNAs identified by CLIP-seq (Fig. 1e and Supplementary Fig. 2b,d). Moreover, we validated the association of SIRT7 with several snoRNAs identified by CLIP-seq in HEK293T cells that stably or transiently express Flag/HA- or Flag-tagged SIRT7 (Supplementary Fig. 2c–f). Moreover, chromatin immunoprecipitation (ChIP) assays revealed that SIRT7 is associated with rDNA and some snoRNA gene loci, for example, *U3* and *U13* snoRNA genes, but not with intron-encoded snoRNA genes, for example, *U14*, *SNORA73A*, *SNORA74A*, *SCARNA10* and *SCARNA13* (Fig. 1f). Together with the observation that expression of U3 snoRNA was decreased by 50% in SIRT7-deficient cells (Supplementary Fig. 2g), this result suggests that SIRT7 affects transcription or stability of U3 snoRNA.

### SIRT7 promotes U3 snoRNA-dependent pre-rRNA processing

**Figure 2:**
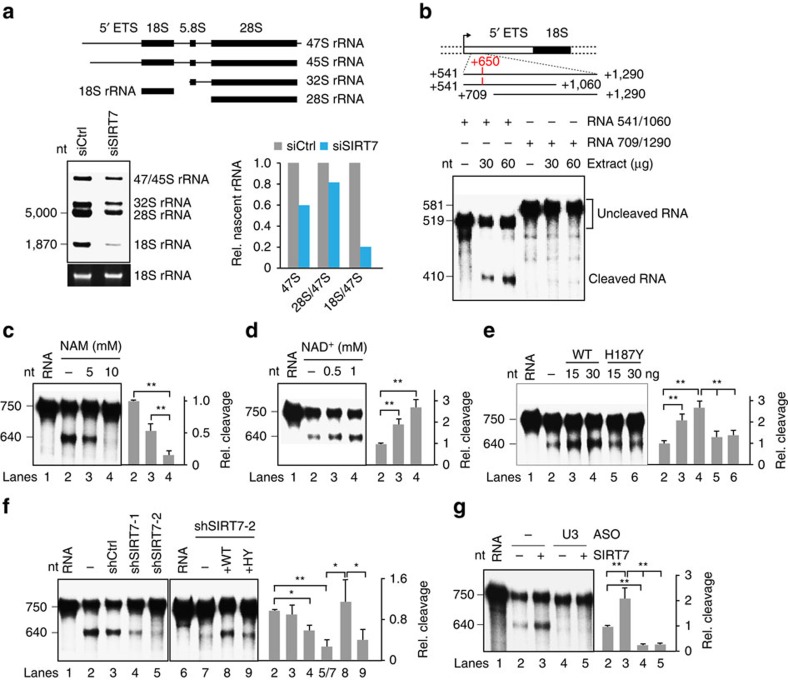
SIRT7 is involved in pre-rRNA processing. (**a**) Knockdown of SIRT7 impairs pre-rRNA synthesis and processing *in vivo*. U2OS cells transfected with control (siCtrl) or SIRT7-specific siRNAs (siSIRT7) were metabolically labelled with ^3^H-uridine. RNA was analysed by agarose gel electrophoresis and fluorography. The bar diagram shows quantification of the processing intermediates, values from siCtrl cells being set to 1. (**b**) *In vitro* processing assay. Extracts from L1210 cells were incubated with ^32^P-labelled RNA comprising the 5′ETS depicted in the scheme above. ^32^P-labelled RNA and cleavage products were analysed by gel electrophoresis and PhosphorImaging. See also Supplementary Fig. 3a. (**c**) 5′ETS processing is inhibited by NAM. The assay contained radiolabelled RNA (+541/+1290) and extracts from L1210 cells cultured for 6 h in the absence or presence of NAM. (**d**) Processing is enhanced by NAD^+^. Processing assays containing radiolabelled RNA (+541/+1290) were substituted with NAD^+^ as indicated. (**e**) The catalytic activity of SIRT7 is required for pre-rRNA cleavage. Assays were supplemented with 15 or 30 ng of purified wildtype (WT) or mutant (H187Y) Flag-SIRT7 (Supplementary Fig. 3b). (**f**) Depletion of SIRT7 impairs processing. SIRT7 was depleted from L1210 cells by shRNAs (shSIRT7-1, shSIRT7-2, Supplementary Fig. 3c). Extracts from non-infected cells (−) or cells expressing control shRNA (shCtrl) served as control (left). To rescue impaired cleavage, 15 ng of wild-type Flag-SIRT7 (WT) or mutant H187Y (HY) were added to SIRT7-depleted extracts (right). (**g**) Depletion of U3 snoRNA abolishes processing. U3 snoRNA was depleted by preincubating extracts with U3-specific antisense oligos (ASO, 50 ng μl^−1^) and 2 U of RNase H (Supplementary Fig. 3d). *In vitro* processing was performed with undepleted (−) or depleted extracts in the absence or presence of 15 ng Flag-SIRT7. Bar diagrams in **c**–**g** show quantification of the ratio of cleaved versus uncleaved transcripts, presented as mean±s.d. from three independent experiments (*P<0.05, **P<0.01, analysis of variance with Bonferroni's test).

The finding that SIRT7 is associated with both pre-rRNA and snoRNAs suggests that beyond its function in rDNA transcription SIRT7 may also be involved in snoRNP-dependent processing of pre-rRNA. To test this, RNA was metabolically labelled in control and SIRT7-deficient cells, and pre-rRNA and processing intermediates were analysed by gel electrophoresis and fluorography (Fig. 2a). Consistent with SIRT7 activating Pol I transcription^25^, depletion of SIRT7 led to roughly 50% reduction in 47/45S pre-rRNA and 28S rRNA. Notably, the level of nascent 18S rRNA was even more decreased, suggesting that SIRT7 plays a role in 18S rRNA processing.

To examine whether SIRT7 promotes U3 snoRNA-dependent cleavage of pre-rRNA within the external transcribed spacer (5′ETS), we performed *in vitro* processing assays using ^32^P-labelled RNA covering the first processing site at position +650. After incubation with extracts from mouse L1210 cells, transcripts were cleaved in a time-dependent fashion, yielding shorter RNAs that were cut at the 5′ETS processing site (Fig. 2b and Supplementary Fig. 3a). A control transcript comprising nucleotides from +709 to +1290 was not cleaved, underscoring the requirement of sequences around the 5′-terminal processing site at +650 for specific RNA cleavage. In support of SIRT7 serving a role in pre-rRNA processing, *in vitro* cleavage of the template RNA was inhibited if extracts were prepared from cells that were treated with nicotinamide (NAM), a competitive inhibitor of sirtuins (Fig. 2c). Conversely, processing activity increased if the reactions were supplemented with NAD^+^ (Fig. 2d), corroborating that the enzymatic activity of sirtuin(s) is beneficial for 5′-terminal processing of pre-rRNA. To prove that SIRT7 is the NAD^+^-dependent enzyme that promotes processing, the assays were performed in the absence or presence of recombinant SIRT7. In accord with SIRT7 promoting pre-rRNA processing, exogenous SIRT7, but not the enzymatically inactive mutant SIRT7/H187Y, enhanced specific cleavage of the template RNA (Fig. 2e and Supplementary Fig. 3b). Moreover, 5′ETS processing was attenuated in extracts from SIRT7-depleted cells, processing being restored after addition of wild-type SIRT7 but not the enzymatically inactive mutant SIRT7/H187Y (Fig. 2f and Supplementary Fig. 3c). Depletion of U3 snoRNA from the cell extract by antisense oligonucleotides abolished cleavage at position +650 regardless of whether SIRT7 was added or not, confirming that *in vitro* processing was dependent on U3 snoRNP (Fig. 2g and Supplementary Fig. 3d). Together, these data demonstrate that both U3 snoRNA and SIRT7 are required for 5′ETS pre-rRNA cleavage, the initial step in 18S rRNA processing.

### SIRT7 counteracts PCAF-dependent acetylation of U3-55k

**Figure 3:**
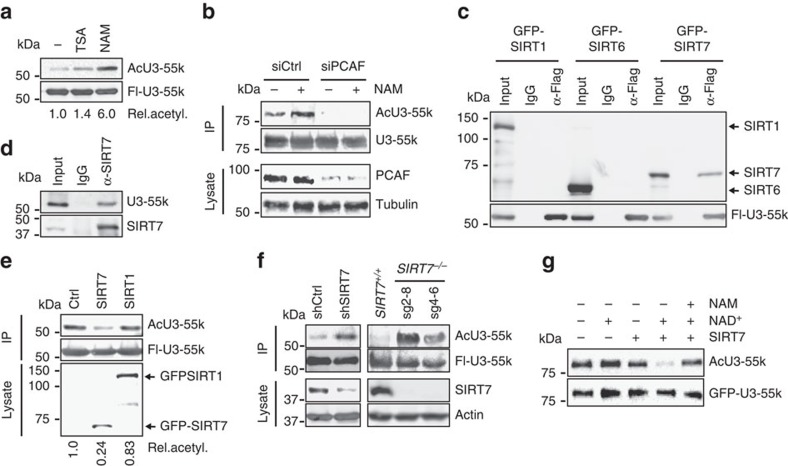
SIRT7 interacts with and deacetylates U3-55k. (**a**) Acetylation of U3-55k is increased on NAM treatment. HEK293T cells expressing Flag-U3-55k were treated for 6 h with 500 nM of TSA, 10 mM of NAM, or were left untreated (−). Acetylation of immunopurified Flag-U3-55k was analysed on western blots using anti-pan-AcK antibody. The blot was reprobed with anti-Flag antibody to monitor the amount of Flag-U3-55k. The numbers below indicate the relative level of acetylation. (**b**) PCAF acetylates U3-55k. HEK293T cells expressing GFP-U3-55k were transfected with siRNA against PCAF (siPCAF) or control siRNA (siCtrl). Acetylation of GFP-U3-55k purified from cells cultured in the absence or presence of NAM (10 mM, 6 h) was detected on western blots with anti-pan-AcK antibody; equal loading of GFP-U3-55k was confirmed by reprobing with anti-GFP (upper panels). Cell lysates were probed with anti-PCAF and anti-tubulin antibodies to demonstrate depletion of PCAF by siRNA (lower panels). (**c**) SIRT7 interacts with U3-55k. Lysates from HEK293T cells co-expressing Flag-U3-55k and GFP-tagged SIRT1, SIRT6 or SIRT7 were incubated with anti-Flag antibody and co-precipitated sirtuins were analysed on western blots with anti-GFP antibodies. (**d**) Association of endogenous SIRT7 with U3-55k. SIRT7 was immunoprecipitated with 5 μg of anti-SIRT7 antibodies and co-precipitated U3-55k was detected on western blots with anti-U3-55k antibodies. (**e**) Overexpression of GFP-SIRT7 decreases acetylation of U3-55k. Flag-U3-55k was immunopurified from HEK293T cells co-expressing GFP-SIRT7, GFP-SIRT1 or Flag-U3-55k alone (Ctrl). Western blots show the level of acetylated Flag-U3-55k probed with anti-pan-AcK antibody, purified Flag-U3-55k was detected with anti-Flag antibody. (**f**) Depletion of SIRT7 leads to hyperacetylation of U3-55k. Western blots monitoring acetylation of Flag-U3-55k from HEK293T cells treated with control or SIRT7-specific shRNA (left panels), or from parental HEK293T cells (*SIRT7*^*+/+*^) and *SIRT7*^*−/−*^ cell lines *sg2–8* and *sg4–6* (right panel; see also Supplementary Fig. 4c). Membranes were probed with antibodies against pan-AcK, Flag, SIRT7 and actin. (**g**) SIRT7 deacetylates U3-55k *in vitro*. Reactions contained 0.8 μg of immunopurified Flag-SIRT7, 2 mM of NAD^+^, or 10 mM of NAM and 1 μg of bead-bound GFP-U3-55k (Supplementary Fig. 4e). Acetylation of GFP-U3-55k was detected with anti-pan-AcK antibody, GFP-U3-55k was analysed with anti-GFP antibody.

Mammalian U3 snoRNPs are composed of the five core subunits U3-55k, NOP56, NOP58, fibrillarin and 15.5k (ref. 4). As the catalytic activity of SIRT7 is required for processing *in vitro*, SIRT7-dependent deacetylation of any core subunit might be required for efficient processing. Previous acetylome studies revealed that the U3 snoRNP-specific protein U3-55k is acetylated in the N-terminal domain^31^. We therefore reasoned that reversible acetylation of this subunit might regulate U3 snoRNA-dependent pre-rRNA processing. To test this, we monitored acetylation of U3-55k protein in cells treated with trichostatin A (TSA), an inhibitor of class I/II HDACs, or with NAM, which specifically inhibits sirtuins. In the absence of any inhibitor or on treatment with TSA, acetylation was barely detectable on western blots using an antibody that recognizes acetylated proteins. However, acetylation was markedly increased if cells were treated with NAM (Fig. 3a) or in cells overexpressing PCAF, suggesting that PCAF is the acetyltransferase that acetylates U3-55k (Supplementary Fig. 4a). In support of this view, short interfering RNA (siRNA)-mediated depletion of PCAF abolished acetylation of U3-55k regardless whether or not cells were treated with NAM (Fig. 3b).

To investigate whether SIRT7 or another nuclear sirtuin deacetylates U3-55k, we monitored the association of U3-55k with SIRT1, SIRT6 and SIRT7. The immunoprecipitation experiments in Fig. 3c,d show that endogenous or Flag-U3-55k was associated with endogenous and ectopic SIRT7, but not with SIRT1 or SIRT6. No binding of U3-55k to SIRT7 was observed if SIRT7 was affinity-purified under denaturing conditions (Supplementary Fig. 4b). In addition, U3-55k acetylation was compromised in cells overexpressing green fluorescent protein-human sirtuin 7 (GFP-SIRT7), but not in cells overexpressing SIRT1, underscoring that SIRT7 targets U3-55k for deacetylation (Fig. 3e). In support of this view, depletion of SIRT7 by short hairpin RNAs (shRNAs) or genetic knockout of *SIRT7* led to hyperacetylation of U3-55k without affecting expression of U3-55k (Fig. 3f and Supplementary Fig. 4c,d). Significantly, recombinant SIRT7 efficiently deacetylated U3-55k *in vitro*, reinforcing that PCAF-mediated acetylation of U3-55k is counteracted by SIRT7 (Fig. 3g and Supplementary Fig. 4e).

### Deacetylation of U3-55k promotes pre-rRNA processing

**Figure 4:**
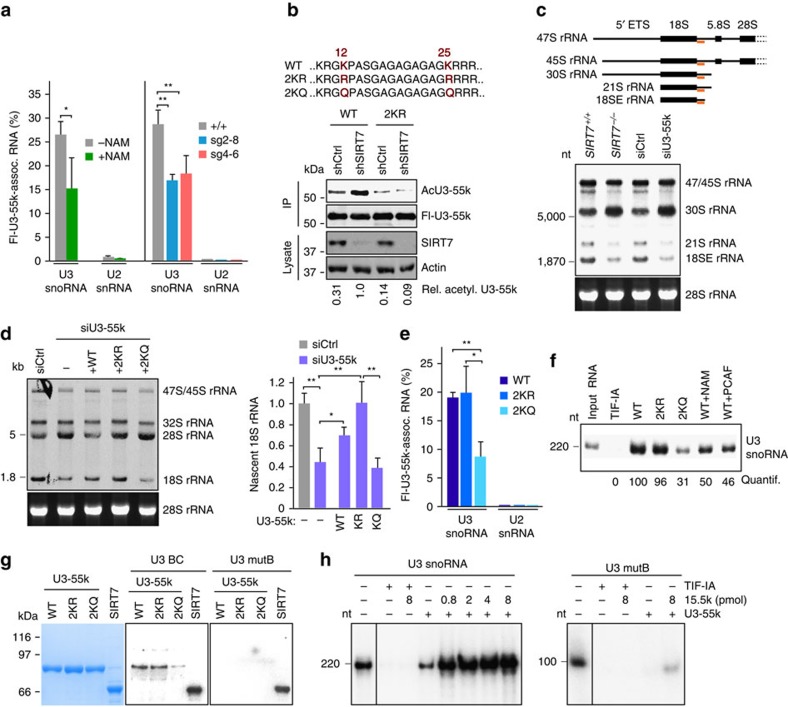
U3-55k deacetylation promotes pre-rRNA processing. (**a**) CLIP-RT–qPCR showing U3 snoRNA binding to U3-55k in HEK293T cells treated for 6 h with 10 mM NAM or not (left panel), and in *HEK293T/SIRT7*^*+/+*^ or *SIRT7*^*−/−*^ clones (*sg2–8* and *sg4–6*). Co-precipitated RNAs were quantified by RT–qPCR. Means±s.d. (*n*=3) are presented (*P<0.05; **P<0.01, analysis of variance (ANOVA) with Bonferroni's test). (**b**) HEK293T cells expressing wildtype (WT) or mutant (2KR) U3-55k were transfected with control (shCtrl) or SIRT7-specific shRNAs (shSIRT7). Acetylation of immunopurified U3-55k was monitored on western blots with anti-pan-AcK antibodies. U3-55k and SIRT7 were detected with anti-Flag and anti-SIRT7 antibody. The lysine residues of U3-55k replaced by arginine (2KR) or glutamine (2KQ) are depicted above. (**c**) Northern blot showing processing intermediates from HEK293T/*SIRT7*^*+/+*^ and *SIRT7*^*−/−*^ cells (clone sg4–6, Supplementary Fig. 4c) and from HEK293T cells transfected with non-targeting (siCtrl) or U3-55k siRNA (Supplementary Fig. 5c,d). Membranes were probed with ^32^P-labelled ITS1 oligo. The position of the ITS1 (orange) hybridization probe is marked in the scheme above. (**d**) Metabolic labelling of nascent RNA in U3-55k-depleted cells. U3-55k-deficient cells were transfected with wild-type or mutant Flag-U3-55k. RNA was pulse-labelled with ^3^H-uridine and analysed by fluorography. Bars represent means of radiolabelled 18S rRNA ±s.d. from three experiments (*P<0.05; **P<0.01). (**e**) Acetylation attenuates RNA binding of U3-55k. CLIP-RT–qPCR showing association of the indicated RNAs with wildtype (WT) or mutant (2KR and 2KQ) Flag-U3-55k. Means±s.d. (*n*=3) are presented (*P<0.05; **P<0.01, ANOVA with Bonferroni's test). (**f**) Hypoacetylation enhances RNA binding of U3-55k. Wild-type or mutant GFP-U3-55k (2KR, 2KQ) were expressed in HEK293T cells in the absence or presence of co-expressed Flag-PCAF. Cells were treated with NAM or not, and bead-bound GFP-U3-55k was incubated with ^32^P-labelled U3 snoRNA. Numbers below show quantification of U3-55k-bound RNA. See also Supplementary Fig. 5h. (**g**) Northwestern blot showing U3-55k binding to U3 snoRNA BC. Indicated proteins were separated by SDS–polyacrylamide gel electrophoresis, blotted onto membranes, and probed with radiolabelled U3 BC or mutant B RNA (ref. 34). (**h**) RNA binding of U3-55k is enhanced by 15.5k. Pull-down of ^32^P-labelled wild-type or box B-mutant (mutB) U3 snoRNA by bead-bound GFP-U3-55k in the absence or presence of GST-15.5k. GFP-TIF-IA served as negative control. See also Supplementary Fig. 5i.

To investigate whether reversible acetylation of U3-55k affects its interaction with U3 snoRNA, we monitored the association of U3-55k with U3 snoRNA by CLIP assays. These experiments revealed that binding of U3-55k to U3 snoRNA inversely correlated with U3-55k acetylation, the interaction with U3 snoRNA being decreased if cells were treated with NAM to inhibit SIRT7 activity (Fig. 4a, left). Consistent with acetylation impairing the interaction between U3-55k and U3 snoRNA, the association of U3-55k with U3 snoRNA was decreased in *SIRT7* knockout cells (Fig. 4a, right). These findings emphasize that deacetylation of U3-55k by SIRT7 is required for efficient binding of U3-55k to U3 snoRNA.

To decipher the functional significance of U3-55k acetylation, we replaced two lysine residues (K12, K25) within the N-terminal domain of U3-55k that were reported to be acetylated^31^ by either arginine or glutamine, and monitored the impact of wild-type and mutant U3-55k on pre-rRNA processing. Overexpression of PCAF increased acetylation of wild-type U3-55k but did not augment acetylation of the K12/K25 mutants, supporting that PCAF acetylates K12 and K25 (Supplementary Fig. 5a). Fluorescence microscopy revealed similar nucleolar/nuclear distribution of wild-type U3-55k and the mutants that mimick the non-acetylated (2KR) or acetylated (2KQ) state of U3-55k, indicating that acetylation of K12/25 did not alter the cellular localization of U3-55k (Supplementary Fig. 5b). Significantly, acetylation of wild-type U3-55k was markedly increased in SIRT7-deficient cells, whereas acetylation of mutant U3-55k remained low, regardless of whether SIRT7 was depleted or not (Fig. 4b).

If deacetylation of U3-55k by SIRT7 is required for proper 18S rRNA processing, depletion of either U3-55k or SIRT7 should cause similar defects in pre-rRNA processing. Indeed, knockdown of U3-55k or knockout of SIRT7 impaired the formation of 18S rRNA processing intermediates 21S and 18SE RNA (Fig. 4c and Supplementary Fig. 5c). This result is in accord with studies showing that U3-55k-dependent cleavage at sites A0 and 1 is required for 18S rRNA processing^10^. The finding that cells depleted of U3-55k or SIRT7 exhibit similar processing defects was further corroborated by metabolic labelling of nascent RNA. The amount of labelled 18S rRNA was markedly reduced in U3-55k-deficient cells, resembling the processing defects observed in SIRT7 knockdown cells (Fig. 4d and Supplementary Fig. 5d). Notably, wild type and the acetylation-deficient mutant of U3-55k (2KR) were capable to rescue the processing defect, whereas the acetylation-mimicking K12/25Q (2KQ) mutant did not restore processing (Fig. 4d and Supplementary Fig. 5e,f). This result is supported by CLIP assays showing that binding of mutant U3-55k/2KQ to U3 snoRNA was compromised, while the acetylation-deficient K12/25R (2KR) mutant bound with similar efficiency as wild-type U3-55k (Fig. 4e and Supplementary Fig. 5g). Likewise, *in vitro* pull-down experiments showed reduced binding of U3 snoRNA to U3-55k/2KQ or to hyperacetylated U3-55k as compared with wild-type U3-55k or the acetylation-deficient mutant U3-55k/2KR (Fig. 4f and Supplementary Fig. 5h).

It is well established that the 15.5k protein interacts with its cognate RNAs via a kink-turn motif. The U3 snoRNA-specific kink-turn motif is comprised in box B/C (refs 32, 33, 34). It was proposed that binding of 15.5k to the B/C motif is essential for recruitment of U3-55k and for the subsequent nucleation of the SSU processome. However, it remained controversial whether U3-55k interacts with U3 RNA independent of the 15.5k protein^34^,^35^. To address this issue, we performed northwestern analyses, incubating membrane-bound U3-55k with radioactive-labelled U3 BC RNA. These experiments revealed that U3-55k and the acetylation-deficient mutant U3-55k/2KR retained U3 snoRNA (U3 BC), while RNA binding of the acetylation-mimicking mutant 2KQ was compromised (Fig. 4g). In contrast, SIRT7 bound with similar efficiency to wild-type and mutant U3 snoRNA (ref. 34) confirming the specificity of U3-55k binding to U3 snoRNA. In complementary pull-down experiments, we tested the interaction of bead-bound GFP-U3-55k with U3 snoRNA. For this, immobilized U3-55k protein (or TIF-IA as a negative control) was incubated with radiolabelled wild-type or mutant U3 RNA, in the absence or presence of increasing amounts of GST-15.5k protein, and bound RNA was visualized by gel electrophoresis and PhosphorImaging. In accord with previous reports^34^,^35^, this assay revealed that binding of U3-55k to U3 snoRNA is markedly stimulated by the 15.5k protein (Fig. 4h and Supplementary Fig. 5i). Again, U3-55k did not bind to mutant U3 (mutB) RNA, regardless whether or not 15.5k was present. Together, these results demonstrate that acetylation impairs the interaction of U3-55k with U3 snoRNA, which in turn is a prerequisite for the production of 18S rRNA. Apparently, SIRT7 keeps U3-55k in the deacetylated state in proliferating cells with high rRNA synthetic activity, allowing association with U3 snoRNA, processome formation and pre-rRNA cleavage.

### Acetylation of U3-55k impairs rRNA processing under stress

**Figure 5:**
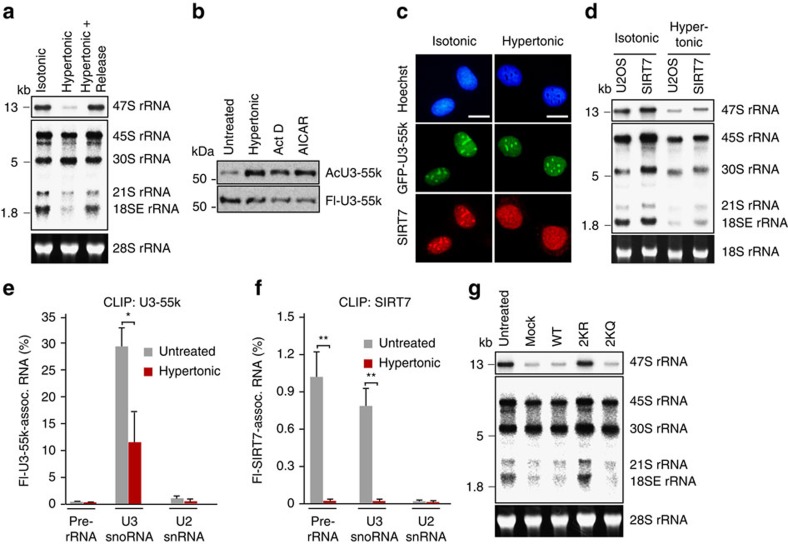
Pre-rRNA transcription and processing are attenuated under stress. (**a**) Northern blot of pre-rRNA and processing intermediates from HEK293T cells that were untreated, exposed to hyperosmotic stress for 90 min (hypertonic), or recovered to regular medium for 60 min (hypertonic rel.). Membranes were probed with ^32^P-labelled antisense riboprobe specific to 47S pre-rRNA (5'ETS, top) or with ITS1 oligos hybridizing to pre-rRNA intermediates (middle panel). (**b**) Acetylation of U3-55k is increased on different cellular stress conditions. HEK293T cells expressing Flag-U3-55k were treated with actinomycin D (Act D, 0.1 μg ml^−1^, 4 h), AICAR (0.5 mM, 12 h) or exposed to hypertonic stress. Acetylation of immunopurified Flag-U3-55k and equal loading was monitored on western blots using anti-pan-AcK and anti-Flag antibodies. (**c**) Cellular localization of SIRT7 and U3-55k on hyperosmotic stress. Images showing localization of GFP-U3-55k and SIRT7 in normal conditions and on exposure to hyperosmotic stress for 90 min. Nuclei were stained with Hoechst 33342. Scale bars, 10 μm. (**d**) Overexpression of SIRT7 alleviates processing defects on hypertonic stress. Northern blot of RNA from parental U2OS cells and from cells which stably express GFP-SIRT7 (U2OS-GFP-SIRT7) using 5′ETS and ITS1 probes as in **a**. (**e**) CLIP-RT–qPCR monitoring binding of Flag-U3-55k to pre-rRNA, U3 snoRNA and U2 snRNA in HEK293T cells cultured in normo-osmotic medium or exposed to hypertonic stress for 90 min. Precipitated RNA was analysed by RT–qPCR using the indicated primers. Bars represent the means±s.d. from three biological repeats (*P<0.05, analysis of variance (ANOVA) with Bonferroni's test). (**f**) CLIP-RT–qPCR of Flag-SIRT7 from HEK293T cells as described under **e**. Co-precipitated RNA was analysed by RT–qPCR using the indicated primers. Bars represent the means±s.d. from three biological repeats (**P<0.01, ANOVA with Bonferroni's test). (**g**) Overexpression of mutant U3-55k-K12/25R rescues processing defects on stress. Northern blot of RNA from HEK293T cells expressing wild-type or mutant Flag-U3-55k exposed to hypertonic stress for 90 min. RNA was hybridized to ^32^P-labelled 5′ETS and ITS1 probes.

To examine the functional relevance of SIRT7-dependent regulation of pre-rRNA processing, we compared levels of pre-rRNA and processing intermediates in cells grown in normal medium and in cells exposed to hypertonic conditions. Northern blot analysis revealed that the 47S precursor was barely detectable on hypertonic stress. The level of 47S pre-rRNA was restored on reversal to normo-osmotic conditions, implying that stress-induced transcriptional repression is reversible (Fig. 5a and Supplementary Fig. 6a). Notably, in hypertonic cells the precursors of 18S rRNA, that is, 21S and 18SE rRNA, were barely detectable, indicating that exposure to hypertonic stress impaired the production of mature 18S rRNA (Fig. 5a).

To decipher the molecular mechanism underlying stress-dependent inhibition of pre-rRNA processing, we monitored U3-55k acetylation under different stress conditions that inhibit nucleolar transcription such as hypertonic stress, treatment with actinomycin D or treatment with 5-aminoimidazole-4-carboxamide ribonucleotide (AICAR). Both physiological and drug-induced stress caused robust hyperacetylation of U3-55k, whereas acetylation of mutant K12/25R (2KR) was not affected (Fig. 5b and Supplementary Fig. 6b). Stress-induced hyperacetylation had little effect on the nucleolar localization of U3-55k, whereas both endogenous and ectopic SIRT7 translocated from nucleoli to the nucleoplasm (Fig. 5c and Supplementary Fig. 6c). These results imply that translocation into the nucleoplasm separates SIRT7 from nucleolar U3-55k, which in turn leads to hyperacetylation of U3-55k and processing defects.

To examine whether stress-induced redistribution of SIRT7 from the nucleolus into the nucleoplasm correlates with compromised pre-rRNA processing, we monitored 18S rRNA processing intermediates in parental U2OS cells and in U2OS cells which stably express GFP-SIRT7. Overall, 47S rRNA levels and relevant processing intermediates were elevated in U2OS/GFP-SIRT7 cells compared with parental U2OS cells (Fig. 5d). On exposure to hypertonic stress, pre-rRNA synthesis and processing were suppressed in both cell lines. However, attenuation of processing was less pronounced in cells expressing GFP-SIRT7. This is probably due to the fact that a fraction of ectopic GFP-SIRT7 remained in nucleoli (Supplementary Fig. 6c), which alleviates processing defects. This result underscores the impact of SIRT7 on pre-rRNA processing, release from nucleoli under stress conditions leading to hyperacetylation of U3-55k and impaired pre-rRNA cleavage.

As U3 snoRNPs are required for 18S rRNA processing and acetylation of U3-55k compromises its interaction with U3 snoRNA, the association of U3-55k with U3 snoRNA should be decreased in cells exposed to hypertonicity. Indeed, CLIP experiments revealed a significant decrease in U3-55k-associated U3 snoRNA in stressed cells, while the overall level of U3 snoRNA remained unaffected (Fig. 5e and Supplementary Fig. 6d). Consistent with SIRT7 being released from nucleoli on transcriptional or energetic stress^24^, binding of SIRT7 to snoRNAs and pre-rRNA was no longer detected under hyperosmotic conditions, supporting that the interaction between SIRT7 and U3 snoRNA was abolished (Fig. 5f). Notably, overexpression of the acetylation-deficient mutant U3-55k/2KR, but not the acetylation-mimicking mutant U3-55k/2KQ, rescued the stress-induced processing defect, emphasizing the functional importance of hypoacetylated U3-55k in pre-rRNA processing (Fig. 5g).

## Discussion

Recent studies have linked expression of SIRT7 to cell proliferation and oncogenic activity, connecting SIRT7-dependent regulation of ribosome biogenesis with checkpoints controlling cell cycle progression, tumorigenesis and formation of metastatic phenotypes^26^,^27^,^36^,^37^,^38^,^39^. Despite this important functional link, the enzymatic activity, the molecular targets and physiological functions of SIRT7 are poorly defined. Similar to other sirtuins, SIRT7 depletion did not globally change the acetylation levels of nucleolar and nuclear proteomes^36^,^40^, indicating that sirtuins exhibit a weak or substrate-specific deacetylase activity. SIRT7 was shown to target lysine 18 of histone H3 (H3K18), hypoacetylation of H3K18 compromising transcription of target genes^36^,^39^. No measurable deacetylase activity of SIRT7 has been detected on other histone peptide substrates *in vitro*, supporting that SIRT7 functions as a highly specific deacetylase. *In vivo,* SIRT7 has been shown to deacetylate GABPβ1, a key regulator of mitochondrial functions, deacetylation of GABPβ1 promoting mitochondrial gene expression^41^. Moreover, SIRT7 deacetylates the Pol I subunit PAF53, which enhances recruitment of Pol I to the rDNA promoter and activates transcription^24^.

In this study, we have identified the nucleolar protein U3-55k as a novel substrate of SIRT7, deacetylation of U3-55k being required for 18S rRNA maturation. U3-55k is associated with U3 snoRNA, the core of the SSU processome, which promotes 5′ETS processing^4^,^7^,^8^,^11^,^42^,^43^. Consistent with pre-rRNA transcription and processing being intertwined, the SSU processome binds to the 5′ ETS of pre-rRNA enabling endonucleolytic cleavage of the primary transcript 650 nucleotides downstream of the 5′ end of pre-rRNA (refs 4, 9, 11, 16). The SSU processome was proposed to act as a pre-rRNA chaperone enabling pre-18S rRNA folding and processing through base pairing of U3 snoRNA with pre-rRNA (refs 11, 14). These findings indicate that the sequence and/or structural elements within the 5′ETS contribute to the accuracy or even the order of processing steps. U3 snoRNA-dependent cleavage of pre-rRNA has been observed in several organisms, including humans and mice^4^,^16^,^29^,^44^, indicating that this mechanism is conserved among eukaryotes.

Although the importance of snoRNAs in pre-rRNA processing and posttranscriptional 2′-O-methylation and pseudouridylation of the rRNAs has been known for decades, our understanding of the molecular mechanisms by which mammalian pre-rRNA processing is regulated is very limited, owing mainly to the lack of *in vitro* systems that respond to regulatory factors. Processing of the 5′ ETS was first linked to snoRNAs in vertebrates when a crude *in vitro* system was shown to faithfully cleave the 5′ ETS in a U3 snoRNA-dependent fashion^5^. Using this system, we found that cleavage of a template RNA bearing the first processing site of mouse pre-rRNA was compromised if SIRT7 activity was inhibited by NAM, or if extracts from SIRT7-depleted cells were used in the processing reactions. Supplementation with NAD^+^ and ectopic SIRT7 stimulated RNA cleavage, whereas an enzymatically inactive SIRT7 mutant failed to rescue processing activity. Moreover, in SIRT7-deficient cells the level of metabolically labelled 18S rRNA was markedly reduced, reinforcing the functional importance of SIRT7-dependent deacetylation of component(s) of the U3 snoRNP in early pre-rRNA processing events.

Our results reveal that SIRT7 regulates pre-rRNA processing by deacetylation of U3-55k, a U3 snoRNA-associated protein that is not present in other box C/D snoRNPs. Consistent with acetylation affecting protein functions, we found that acetylation by PCAF impairs binding of U3-55k to U3 snoRNA, the association of U3-55k with U3 snoRNA being required for processing. Deacetylation by SIRT7, on the other hand, facilitates the interaction of U3-55k with U3 snoRNA, thus promoting pre-rRNA processing. The intimate link between SIRT7 activity and acetylation-dependent U3-55k function is underscored by the finding that knockdown of U3-55k led to the same defects in pre-rRNA processing as those observed on knockout of SIRT7. Overexpression of wild-type U3-55k but not the acetylation-mimicking mutant 2KQ overcame the processing defects, emphasizing that SIRT7 and U3-55k are functionally connected.

The link between U3-55k acetylation and its removal by SIRT7 not only highlights the central role of SIRT7 in ribosome biogenesis but also points to an active role of SIRT7 as a pro-survival adaptor molecule in conditions of cellular stress^24^,^45^,^46^,^47^. Previous studies have demonstrated that SIRT7 is released from nucleoli in response to transcriptional or metabolic stress, leading to hyperacetylation of the Pol I-associated factor PAF53 and downregulation of rDNA transcription^24^. The present study shows that translocation of SIRT7 on short exposure to hyperosmotic stress leads also to hyperacetylation of U3-55k and hence inhibition of pre-rRNA processing. Nucleolar retention of overexpressed SIRT7 under hypertonic stress attenuates processing defects corroborating the stress tolerance conferred by SIRT7 to the cells. Hyperosmolarity impaired the generation of 21S and 18SE pre-rRNA, whereas 30S pre-RNA was hardly affected. Interestingly, RNAi-mediated knockdown of U3-55k or other U3 snoRNA-specific proteins, such as NOP56, NOP58 or fibrillarin, led to the same processing defects as hyperosmolarity^10^, supporting that SIRT7 regulates key factors involved in rDNA transcription and pre-rRNA processing. Thus SIRT7 plays a dual function in ribosome biogenesis, coupling rDNA transcription and pre-rRNA processing by deacetylating PAF53 and U3-55k. Nucleolar release of SIRT7 in response to environmental or metabolic cues disrupts this crosstalk, enhancing acetylation of PAF53 and U3-55k, which in turn inhibits Pol I transcription and processing. Given that SIRT7 interacts with many RNAs that are transcribed by Pol II, it is tempting to speculate that SIRT7 function is not restricted to rDNA but might also prompt other genes to promote cellular survival and stress resistance, thus regulating a variety of important cellular processes.

## Methods

### Transfections and cell treatments

U2OS and HEK293T cells (ATCC) cultured in DMEM/10% foetal bovine serum were transfected with expression vectors encoding epitope-tagged proteins using the calcium phosphate precipitation technique or Fugene6 (Life Technologies). Cells were harvested 36–48 h post transfection. To generate clonal cell lines that stably express FLAG/HA- or GFP-tagged SIRT7, cells were selected in the presence of G418 (750 μg ml^−1^). siRNAs against SIRT7 (hSIRT7 ON-TARGETplus SMARTpool), PCAF (hPCAF ON-TARGETplus SMARTpool), or non-targeting control siRNAs were from Dharmacon (ThermoFisher Scientific) and siRNAs against U3-55k (Supplementary Table 2) from Life Technologies. After reverse transfection with Lipofectamine 2000 or RNAiMax (Invitrogen), cells were harvested after 48–60 h. pLKO.1 plasmids containing mouse SIRT7-specific shRNAs were co-transfected with Pax-2 and VSV-G plasmids into HEK293T cells for production of Lentiviruses. Lentivirus-mediated depletion of SIRT7 was performed in L1210 cells (ATCC) cultured in DMEM/10% horse serum. To induce hyperosmotic stress, cells were cultured for 60–90 min in medium supplemented with 200 mM NaCl. Transcriptional and metabolic stress was induced by treatment with actinomycin D (100 ng ml^−1^, 4 h) and AICAR (0.5-1 mM, 12 h), respectively.

### Plasmids and antibodies

Plasmids encoding hSIRT7, sh-hSIRT7, Flag-PCAF, CBP-HA and p300-HA have been described^25^,^48^. To generate GFP- and Flag-tagged U3-55k, cDNA encoding human U3-55k (NCBI reference sequence: NM_004704.4) was inserted into pEGFP-C (Clontech) or pCMV-Tag2 (Stratagene). Point mutations converting K12 and K25 of U3-55k into arginine (2KR) and glutamine (2KQ) were introduced by PCR. To generate FLAG/HA-tagged hSIRT7, the sequence of the Flag/HA-tag was amplified by PCR and cloned into the plasmid pCMV-hSIRT7. Plasmids encoding mSIRT7-specific shRNAs were generated by cloning the corresponding sequences into pLKO.1. Oligos used for plasmid construction and site-directed mutagenesis are listed in Supplementary Table 3. Polyclonal antibodies against SIRT7 were generated in rabbits and purified as described^25^. Briefly, rabbits were immunized with purified GST-SIRT7(1-86) expressed in E. coli, and the antiserum was purified using GST-SIRT7(1-86) coupled to Affigel (BioRad). Commercial antibodies include anti-acetylated lysine (AcK, Cell Signaling, 9441), anti-nucleolin (Santa Cruz, sc-13057), anti-U3-55k (Abcam, ab56460), anti-Flag (Sigma, F3165), anti-actin (Abcam, ab8227), anti-tubulin (Sigma, clone B-5-1-2, T6074), anti-GFP (Abcam, ab290), anti-PCAF (Santa Cruz, sc-8999) and anti-UBF (Santa Cruz, sc-13125). Anti-HA and anti-GST antibodies were a gift from E. Kremmer (Helmholtz Center Munich). The anti-Flag M2 Affinity Gel (Sigma, F1804) was used for precipitation of Flag-tagged proteins and the GFP-Trap (Chromotek, gta) for purification of GFP-tagged proteins.

### Immunoprecipitation of cross-linked RNA

RNA-immunoprecipitation from UV-cross-linked HEK293T cells expressing Flag-SIRT7 or Flag-U3-55k was essentially done as follows^24^. Nuclei from UV-irradiated (254 nm, 0.15 J cm^−2^) cells were lysed in 20 mM Tris-HCl pH 8.0, 200 mM NaCl, 1 mM EDTA, 1 mM EGTA, 0.1% SDS, 1% NP-40, 0.5% sodium deoxycholate in the presence of protease inhibitors (Roche Complete). After sonication, lysates were precleared with protein G Sepharose (1 h, 4 °C), adjusted to 0.05% SDS and 0.25% sodium deoxycholate, and protein-RNA complexes were immunoprecipitated overnight at 4 °C using anti-Flag M2 beads (Sigma) or protein G Sepharose (GE Healthcare) coated with mouse IgG (Dianova) as control. Beads were washed three times in immunoprecipitation (IP) buffer and twice in IP buffer containing 400 mM KCl. After elution with the Flag peptide (20 μg per 100 μl, 4 h) and proteinase K digestion (30 min, 55 °C), RNA was isolated with TRIzol (ThermoFisher Scientific), incubated with DNase I (Sigma), reverse transcribed using random hexamers and M-MLV reverse transcriptase (Applied Biosystems) and cDNA was amplified by qPCR.

For affinity-purification of SIRT7-V5/His under native conditions, nuclei from UV-cross-linked cells (254 nm, 0.15 J cm^−2^) were lysed in buffer containing 50 mM Tris-HCl pH 7.6, 200 mM NaCl, 0.1% NP-40, 5 mM ß-mercaptoethanol and protease inhibitors (Roche Complete). After binding of SIRT7-V5/His to Ni-NTA-agarose (Qiagen), beads were washed with lysis buffer containing 500 mM NaCl. Protein-RNA complexes were eluted with 500 mM imidazole, 50 mM Tris-HCl pH 7.6, 50 mM NaCl, 0.1% NP-40, 5 mM ß-mercaptoethanol and digested with proteinase K (0.2 mg ml^−1^, 30 min, 55 °C). RNA was isolated with TRIzol, treated with DNase I (Sigma), transcribed into cDNA using random hexamers and M-MLV reverse transcriptase (Life Technologies) and quantified by qPCR using SYBR Green I Master mix (Roche) and Roche Lightcycler 480. The gene-specific primers are listed in Supplementary Table 3. For affinity-purification under denaturing conditions, lysis of nuclei and binding was performed in 50 mM Tris-HCl pH 7.6, 300 mM NaCl, 0.1% NP-40, 5 mM ß-mercaptoethanol, 10 mM imidazole and 6 M guanidinium-HCl. After washing with denaturing buffer and two washes with 50 mM Tris-HCl pH 7.6, 10 mM MgCl_2_, 0.5% NP-40, 10 mM ß-mercaptoethanol protein-RNA complexes were eluted and RNA was monitored by RT–qPCR.

### CLIP-Seq and bioinformatic analysis

The CLIP-seq experiments were performed in biological duplicates. Co-immunoprecipitated RNA from anti-Flag-SIRT7 and IgG control immunoprecipitations was purified with TRIZOL, and treated with DNase I. The RNA-seq libraries were created using the NEBNext Ultra RNA Library Prep kit for Illumina (E7530) with NEBNext Multiplex Oligos for Illumina (E7300). 50 cycles of sequencing were performed on the Illumina HiSeq 2000 Instrument. cDNA libraries from total RNA were used as Input. The reads were mapped to human hs37 reference genome (including human rRNA sequence) using Tophat2 with default parameters. The reads with mapping quality >0 were used for analysis. The RPKM (reads per kilobase per million mapped reads) values were calculated for all genes with annotation in Gencode V17 including mRNA and snoRNAs. The SIRT7 reads distributions on genes transcribed by different polymerases (Pol I, II and III) were calculated and normalized to the total number of mapped reads. To identify snoRNAs encoded in introns of host genes, snoRNAs coordinates were retrieved from Gencode V17 annotations. The coverage plots were produced with R Gviz package. Gene ontology (GO) annotations were done using DAVID v6.7.

### Generation of SIRT7 knockout cell lines by CRISPR-Cas9

Four single guide RNAs (sgRNAs 1-4) that target different regions within exon 1 of the human SIRT7 gene were selected from previously published genome-wide human sgRNA libraries^49^,^50^. The sequences are given in Supplementary Table 3. Oligos corresponding to the sgRNAs were cloned into the lentiCRISPRv1 vector containing the *SpCas9* gene and a puromycin selection marker gene^50^,^51^. HEK293T cells were transfected with either of the sgRNAs and selected with puromycin (750 ng ml^−1^) 24 h post transfection. Single clones were retrieved after 7 days of puromycin selection, expanded and analysed for abrogation of SIRT7 expression by western blotting. Manifestation of the *SIRT7* mutations was verified by PCR and sequencing.

### Metabolic labelling of nascent RNA

0.5 × 10^6^ U2OS or HEK293T cells were cultured for 3 h in growth medium containing 5 μCi ^3^H-uridine (PerkinElmer). Total RNA (2 μg) was separated on a denaturing 1% agarose gel in 20 mM MOPS, pH 7.0, 5 mM sodium acetate and 1 mM EDTA, transferred to Hybond-N^+^ nylon membranes (GE Healthcare), and labelled RNA was visualized by fluorography using EN^3^HANCE (PerkinElmer).

### Chromatin immunoprecipitation

ChIP experiments were performed as follows^25^. In brief, nuclei were isolated from HEK293T or U2OS cells fixed with 1% formaldehyde (10 min, room temperature) and quenched with 0.125 M glycine. After sonication (Bioruptor, Diagenode) to yield 250–500 bp fragments chromatin was diluted fivefold with IP dilution buffer (0.01% SDS, 1.1% Triton X-100, 1.2 mM EDTA, 16.7 mM Tris-HCl pH 8.0 and 167 mM NaCl), precleared with protein A and G Sepharose (GE Healthcare) in the presence of 200 μg ml^−1^ of sonicated *E. coli* DNA, and incubated overnight with the respective antibodies. Protein-DNA complexes were captured on protein A and G Sepharose followed by washes in low-salt buffer (150 mM NaCl, 50 mM Tris-HCl pH 8.0, 5 mM MgCl_2_, 1% Triton X-100), high-salt buffer containing 500 mM NaCl, LiCl buffer (250 mM LiCl, 10 mM Tris-HCl pH 8.0, 5 mM EDTA, 0.5% Na-deoxycholate and 0.5% Triton X-100) and Tris EDTA buffer. After reversal of the cross-link, DNA was purified and amplified by qPCR. Precipitated DNA was calculated as the percentage of input DNA. Primers are listed in the Supplementary Table 3.

### *In vitro* processing assay

Processing of pre-rRNA *in vitro* was performed as follows^5^. Extract proteins (60–80 μg) from L1210 cells were incubated with 20 fmoles of ^32^P-labelled synthetic transcripts in 20 mM Hepes pH 7.9, 2 mM MgCl_2_, 0.14 mM EDTA, 1.5 mM ATP, 120 mM KCl, 2 mM DTT and 9% (v/v) glycerol. The reaction was terminated by the addition of an equal volume of 2% SDS, 12.5 mM EDTA, 0.4 mg ml^−1^ glycogen and 0.4 mg ml^−1^ proteinase K. After incubation for 30 min at 42 °C, radiolabelled RNA was analysed by electrophoresis on 6% polyacrylamide/TBE gels and PhosphorImaging (Fujifilm).

### Co-immunoprecipitation

Cells were lysed in 20 mM Tris-HCl pH 7.9, 0.1% NP-40, 150 mM KCl, 5 mM MgCl_2_, 0.2 mM EDTA, 10% (v/v) glycerol and Complete protease inhibitors (Roche). Cleared lysates were incubated overnight with the respective antibodies or control IgGs, precipitated proteins were eluted with the corresponding epitope peptides or with SDS sample buffer and visualized on western blots.

### Western blotting

Western blotting was performed according to standard procedures. Antibodies used were anti-SIRT7 (1:2000), anti-HA (1:1,000), anti-Flag (1:1,000), anti-actin (1:5,000), anti-tubulin (1:5,000), anti-acetyl-lysine (1:500), anti-PCAF (1:500), anti-U3-55k (1:1,000), anti-GFP (1:10,000), anti-nucleolin (1:1,000), anti-GST (1:2,000) and anti-rabbit or anti-mouse secondary antibodies conjugated to HRP (1:20,000, Dianova 111-035-144, and 115-035-062). Signals were visualized using LAS-3000 (Fujifilm) and ImageGauge. Uncropped blots are shown in the Supplementary Fig. 7.

### *In vitro* deacetylation

HEK293T cells co-expressing GFP-U3-55k and Flag-PCAF were harvested and lysed in buffer AM-400 (400 mM KCl, 20 mM Tris-HCl pH 8.0, 0.2 mM EDTA pH 8.0, 5 mM MgCl_2_ and 10% (v/v) glycerol) supplemented with 0.1% NP-40, Roche Complete protease inhibitors, 500 nM TSA, and 10 mM NAM, and GFP-U3-55k was purified using GFP-Trap. Flag-SIRT7 was immunopurified from HEK293T cells. In brief, cells were lysed in buffer AM-300 (300 mM KCl, 20 mM Tris-HCl pH 7.9, 5 mM MgCl_2_, 0.2 mM EDTA, 10% glycerol and 0.5 mM DTT) supplemented with 0.1% NP-40, protease inhibitors (Roche Complete), and HDAC inhibitors (500 nM TSA, 5 mM sodium butyrate and 10 mM NAM). Lysates were incubated overnight at 4 °C with M2 anti-Flag beads (Sigma). After washing, tagged proteins were eluted in buffer AM-300 supplemented with 0.1% NP-40 and Flag peptide (20 μg per 100 μl). For *in vitro* deacetylation, 0.8–1 μg of pre-acetylated bead-bound-GFP-U3-55k was incubated for 1 h at 30 °C with 0.8 μg of Flag-SIRT7 in 10 mM Tris-HCl pH 8.0, 4 mM MgCl_2_, 0.2 mM DTT, 10% glycerol in the presence or absence of 2 mM NAD^+^. Acetylation of U3-55k was monitored on immunoblots using anti-pan-AcK antibodies.

### Immunofluorescence

Cells grown on poly-L-lysine coated coverslips (ThermoFisher Scientific) were fixed for 10 min with 2.4% paraformaldehyde, permeabilized for 30 s with ice-cold methanol and incubated overnight at 4 °C with anti-UBF (Santa Cruz cat. no. sc-13125, 1:600). For visualization of SIRT7, cells were permeabilized for 10 min with PBS containing 0.5% Triton X-100 and incubated overnight with purified anti-SIRT7 antibody (1:200). Alexa Fluor555-labelled secondary antibody (Life Technologies, 1:400) was used in indirect immunofluorescence and nuclei were counterstained with 5 μg ml^−1^ of Hoechst 33342 (Sigma). Images were processed using NIS-Elements BR 3.10 and ImageJ software.

### RNA analysis

RNA was isolated with TRIzol Reagent (Invitrogen), transcribed into cDNA using random primers (Roche), and analysed by real-time PCR (Roche, LightCycler480) using primers listed in Supplementary Table 3. Alternatively, 47S pre-rRNA was assayed on northern blots by hybridization with a ^32^P-labelled antisense RNA probe complementary to the first 155 nucleotides of pre-rRNA^25^. Processing intermediates were monitored by hybridization to a ^32^P-labelled ITS1 oligonucleotide (Supplementary Table 3). The membrane was pre-hybridized in 6xSSC, 5xDenhardt's, 0.5% SDS and 100 μg ml^−1^ transfer RNA for 1 h at 45 °C, followed by hybridization in the same buffer supplemented with the ^32^P-oligo probe for 16 h at 45 °C. After washing at room temperature in 2xSSC, 0.1% SDS (twice for 10 min) and in 1xSSC, 0.1% SDS (10 min) radioactive signals were visualized by PhosphorImaging.

### RNA pull-down experiments

Immobilized GFP-tagged U3-55k was incubated for 3 h at 4 °C with radiolabelled U3 snoRNA or mutB RNA (ref. 34) in buffer AM-200 (200 mM KCl, 20 mM Tris-HCl pH 8.0, 0.2 mM EDTA, 5 mM MgCl_2_, 10% (v/v) glycerol, 0.1% NP-40, 1 mM EGTA and 10 mM ß-mercaptoethanol). After stringent washing, bound RNA was extracted, subjected to gel electrophoresis and visualized by PhosphorImaging.

### Northwestern blot

Northwestern assays were performed as described^24^. Briefly, purified proteins were separated by SDS-polyacrylamide electrophoresis, transferred to nitrocellulose membranes (GE Healthcare) and proteins were re-natured in 10 mM Tris-HCl pH 6.8, 25 mM NaCl, 1 mM EDTA, 0.04% BSA and 0.04% NP-40 at 4 °C (16 h) and incubated for 2 h at room temperature in the same buffer supplemented with 6.6 nM of ^32^P-labelled *in vitro* transcribed U3 snoRNA. After washing in buffer containing 150 mM NaCl, 50 mM Tris-HCl pH 8.0 and 0.1% Tween-20, bound RNA was detected by PhosphorImaging.

### Statistics and quantitative analyses

The values in the graphs show means of three independent experiments with error bars representing s.d. SPSS 16.0 software was used for statistical analysis. Analyses of variance were performed using analysis of variance with Bonferroni's test. The significance level was set at *P*<0.05 or *P*<0.01. Quantification of western blot signals and radioactive signals was performed using ImageJ and Image Gauge software, respectively.

## Additional information

**Accession codes:** The CLIP-seq data generated in this study have been deposited into Gene Expression Omnibus data base hosted at the NCBI under the accession code GSE76578.

**How to cite this article:** Chen, S. *et al.* SIRT7-dependent deacetylation of the U3-55k protein controls pre-rRNA processing. *Nat. Commun.* 7:10734 doi: 10.1038/ncomms10734 (2016).

## Supplementary Material

Supplementary Information Supplementary Figures 1-7, Supplementary Tables 1-3 and Supplementary References
