# Supplementary material for: SIRT7-dependent deacetylation of the U3-55k protein controls pre-rRNA processing
Source: Nat Commun. 2016 Feb 12;7:10734. doi: 10.1038/ncomms10734 (PMC4754350; doi:10.1038/ncomms10734)
Supplement: Supplementary Information — Supplementary Figures 1-7, Supplementary Tables 1-3 and Supplementary References [file ncomms10734-s1.pdf]

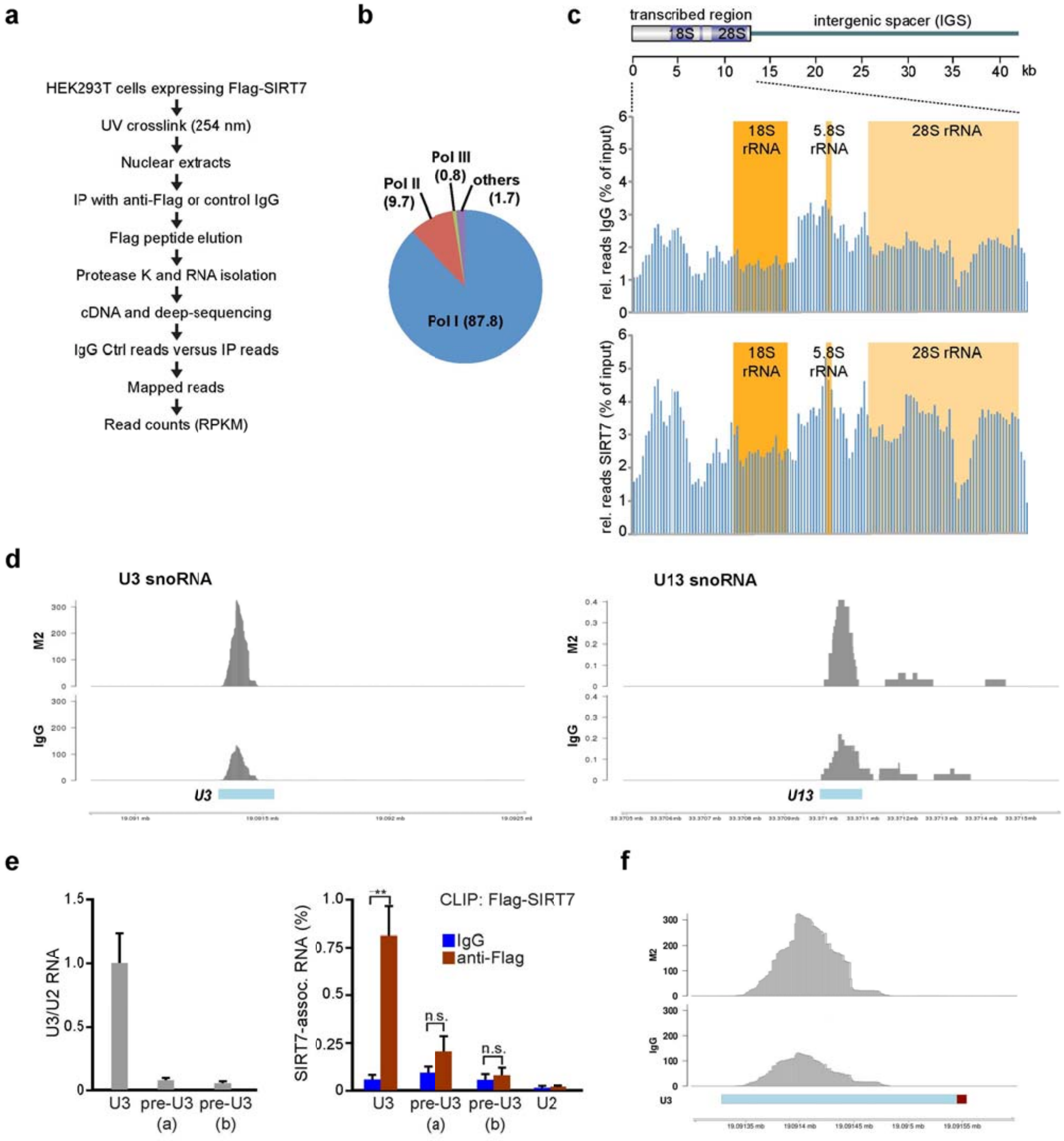

Supplementary Figure 1. SIRT7 CLIP-seq data analysis.

(**a**) Experimental pipeline for SIRT7 CLIP-seq. RPKM: Reads Per Kilobase of transcript per Million mapped reads. (**b**) SIRT7 CLIP-seq reads annotated to Pol I (pre-rRNA), Pol II or Pol III transcribed RNAs of the human genome. The percentage distribution of each to the total reads is indicated. Numbers in brackets indicate the percentage of reads annotated to Pol I, II, or III transcripts, respectively. Others comprise RNAs from pseudogene, lincRNA and 3'-overlapping ncRNA. (**c**) SIRT7 and IgG CLIP-seq reads mapped to human rDNA. The region encoding 18S, 5.8S and 28S rRNA is highlighted. The SIRT7 (lower panel) or IgG (upper panel) reads were normalized to input reads. The y-axis indicates the percentage to input. (**d**) Distribution of SIRT7 reads over U3 and U13 snoRNA genes. Browser view of reads from Flag-SIRT7 associated RNAs (IgG, control IgG; M2, anti-Flag-SIRT7) with ENSEMBL transcript annotation tracks shown below the x-axis. The blue line indicates the position of the U3 and U13 RNA gene locus, respectively; values at the y-axis represent normalized read counts (RPKM). (**e**) SIRT7 is associated with U3 snoRNA but not with pre-U3 snoRNA. Flag-SIRT7 CLIP was

performed in HEK293T cells stably expressing Flag/HA-SIRT7. IgG was used as negative control (right panel). The relative expression levels of U3 snoRNA and pre-U3 RNA (left panel) were detected by RT-qPCR using two different reverse primers (a, b) for detection of pre-U3 snoRNA. The y-axis (left panel) indicates the rel. U3 snoRNA normalized to U2 snRNA. SIRT7 associated U3 and pre-U3 snoRNAs were analyzed by CLIP-RT-qPCR (right panel). Bars represent means ±s.d., n=3 (**$P < 0.01$). (**f**) Distribution of SIRT7 CLIP-seq reads at the U3 snoRNA gene. Browser view of reads from Flag-SIRT7 associated RNAs (IgG, control IgG; M2, anti-Flag-SIRT7) with ENSEMBL transcript annotation tracks shown below the x-axis. The blue line indicates the position of the U3 snoRNA gene, the red line indicates the 8 nt extension present at pre-U3 snoRNA.

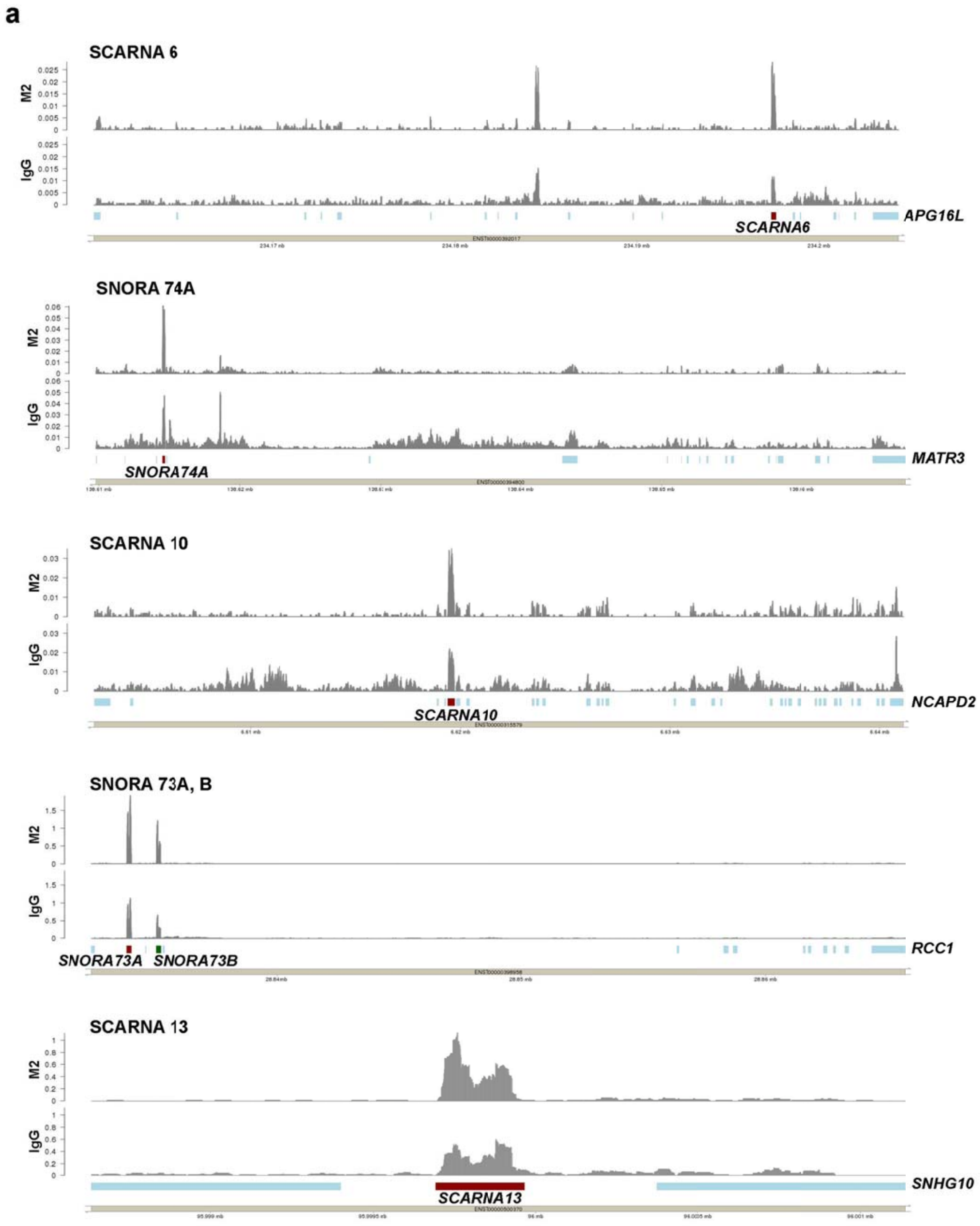
a
SCARNA 6
M2
IgG
APG16L
SCARNA6
SNORA 74A
M2
IgG
MATR3
SNORA74A
SCARNA 10
M2
IgG
NCAPD2
SCARNA10
SNORA 73A, B
M2
IgG
RCC1
SNORA73A
SNORA73B
SCARNA 13
M2
IgG
SNHG10
SCARNA13

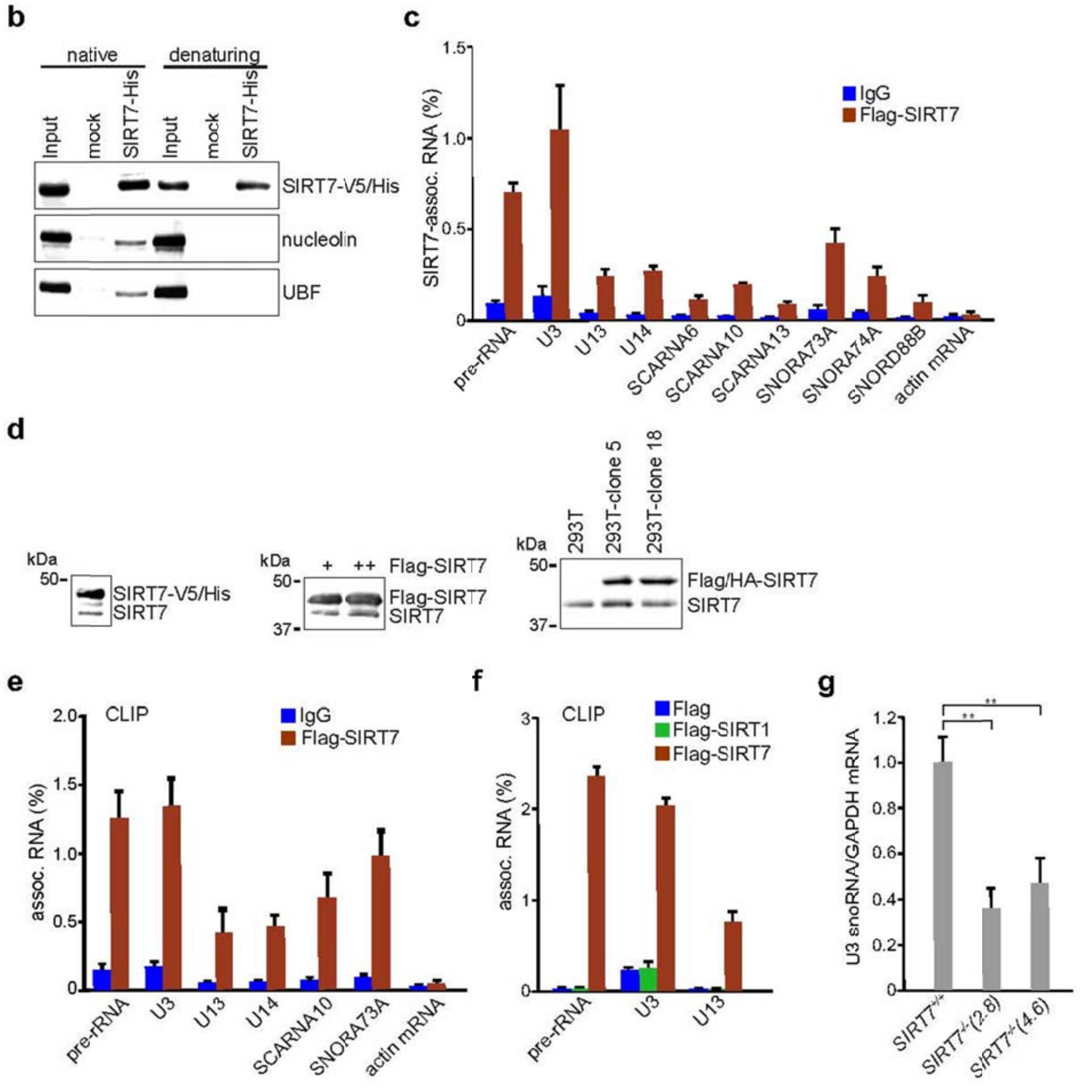

Supplementary Figure 2. **Validation of selected SIRT7 CLIP-seq hits.**

(**a**) Distribution of SIRT7 reads at host genes of selected intron-encoded snoRNAs. Browser view of reads from Flag-SIRT7 associated RNAs (IgG, control IgG; M2, anti Flag-SIRT7) with ENSEMBL transcript annotation tracks shown below the x-axis. The blue lines indicate the position of the exons of the host gene, the red or green lines the position of the snoRNA gene loci. The y-axis represents RPKM. (**b**) The interaction of SIRT7 with nucleolin and UBF is abolished under denaturing conditions. HEK293T cells expressing V5/His-tagged SIRT7 (SIRT7-V5/His) or untransfected (mock) cells were lysed in non-denaturing or denaturing buffer, and His-tagged SIRT7 was affinity-purified under native or denaturing conditions. Co-purification of nucleolin and UBF was monitored on western blots. See also **Fig. 1g**. (**c**) Validation of SIRT7 CLIP-seq data using HEK293T cells stably expressing Flag/HA-tagged SIRT7 (clone 5). SIRT7-associated RNAs were monitored by RT-qPCR using primers specific to the indicated RNAs (see **Supplementary Table 3**). 5' ETS of pre-rRNA was amplified by primer H1. Data show means ±s.d., n=3. (**d**) Representative western blots showing the expression levels of SIRT7-V5/His (left) and Flag-SIRT7 (middle) transiently expressed in HEK293T cells, and expression levels of FLAG/HA-SIRT7 in two HEK293T cell clones used in this study. All blots were probed with purified anti-SIRT7 antibodies. (**e**) Validation of SIRT7 CLIP-seq data using HEK293T cells that transiently express Flag-SIRT7. RNAs precipitated by control (IgG) or anti-Flag antibodies (Flag-SIRT7) were quantified by RT-qPCR using primers specific to the indicated RNAs (see **Supplementary Table 3**). Pre-rRNA was amplified by primer H1. Bars show means ±s.d., n=3. (**f**) Validation of SIRT7 CLIP-seq data using

HEK293T cells that were transfected with empty vector (Flag), and vector expressing Flag-SIRT1, or Flag-SIRT7. Co-precipitated RNAs were quantified by RT-qPCR using primers specific to the indicated RNAs (see **Supplementary Table 3**). Pre-rRNA was amplified by primer H1. Bars show means ±s.d., n=3. (**g**) U3 snoRNA is decreased in SIRT7 deficient cells. U3 snoRNA expression was monitored by RT-qPCR in *HEK293T/SIRT7*$^{+/+}$ and knockout cell lines *sg2-8* and *sg4-6*. Bars represent means ±s.d., n=3 (***P*<0.01).

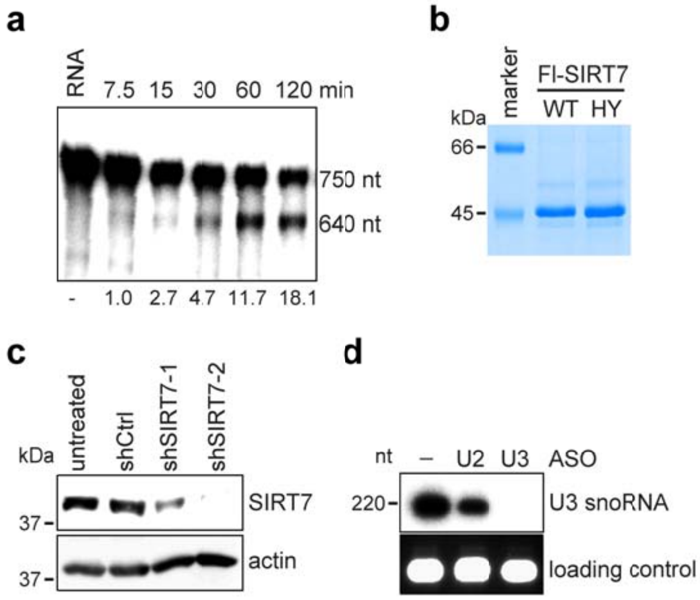

Supplementary Figure 3. SIRT7 regulates early processing events.

(**a**) *In vitro* processing assays using extracts from mouse L1210 cells and ^{32}P-labeled RNA comprising nucleotides from positions +541 to +1290 of mouse pre-rRNA (see **Fig. 2b**). Reactions were terminated after the indicated time and radiolabeled RNA was analysed by gel electrophoresis and PhosphorImaging. Numbers below indicate the ratio of cleaved 640 nt RNA versus uncleaved 750 nt RNA. (**b**) Coomassie blue-stained SDS/PAA gel of purified wildtype Flag-SIRT7 (WT) and the catalytically inactive point mutant (HY). Broad range protein standards were used as marker. (**c**) Knockdown of SIRT7 in mouse L1210 cells by SIRT7-specific shRNAs. Western blot of SIRT7 and actin (loading control) in untreated cells and cells expressing a control shRNA (shCtrl), or two SIRT7-specific shRNAs (shSIRT7-1, shSIRT7-2). (**d**) Depletion of U3-snoRNA by antisense oligonucleotides (ASO). Northern blot showing specific depletion of U3 snoRNA by U3-specific but not by U2 snRNA-specific ASO. An ethidium bromide stain of loaded RNA is shown at the bottom panel.

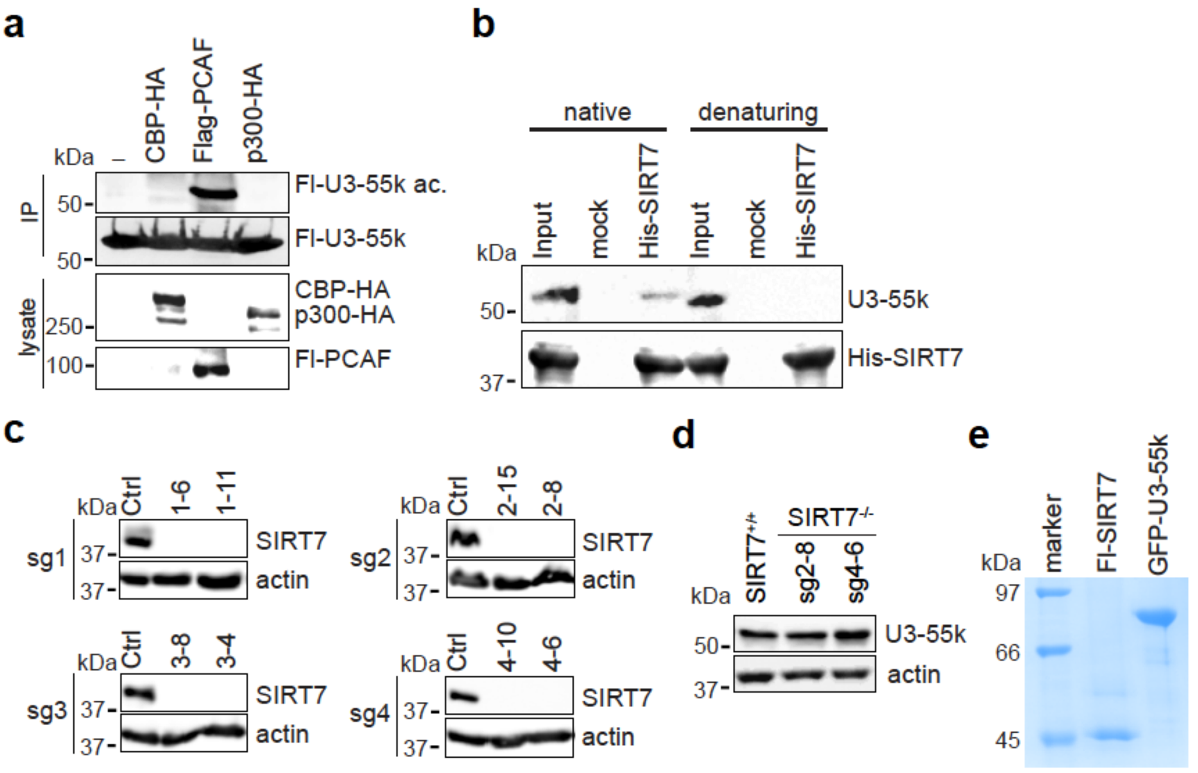

Supplementary Figure 4. **Acetylation and deacetylation of U3-55k is mediated by PCAF and SIRT7 respectively.**

(**a**) PCAF acetylates U3-55k. Flag-U3-55k was co-expressed with either of the indicated acetyltransferases (CBP-HA, Flag-PCAF, or p300-HA) in HEK293T cells. Western blots show immunopurified Flag-U3-55k, probed with anti-pan-AcK and anti-Flag (IP panels). Expression of CBP-HA, p300-HA, and Flag-PCAF was monitored with anti–HA and anti–Flag antibody (lysate panels). (**b**) The interaction of SIRT7 with U3-55k is lost under denaturing conditions. SIRT7-V5/His was affinity-purified from untransfected (mock) HEK293T cells or from cells expressing SIRT7-V5/His using native or denaturing conditions. Co-purification of U3-55k was detected on western blots with anti-U3-55k antibody. (**c**) Western blot showing abrogation of SIRT7 expression in *HEK293T/SIRT7*$^{-/-}$ cell clones generated with 4 different SIRT7-gene specific sgRNAs (sg1-sg4). The control lanes contain lysates from parental *HEK293T/SIRT7*$^{+/+}$ cells (Ctrl). Equal loading was verified by reprobing the membrane with anti-actin. (**d**) Expression of U3-55k is not affected by depletion of SIRT7. Western blot showing levels of U3-55k in two different *SIRT7*$^{-/-}$ cell clones and in parental HEK293T cells (*SIRT7*$^{+/+}$). (**e**) Coomassie blue-stained SDS/PAA gel of Flag-SIRT7 and GFP-U3-55k purified from HEK293T cells used for the *in vitro* deacetylation assays shown in **Fig. 3g**. Broad range protein standards were used as marker.

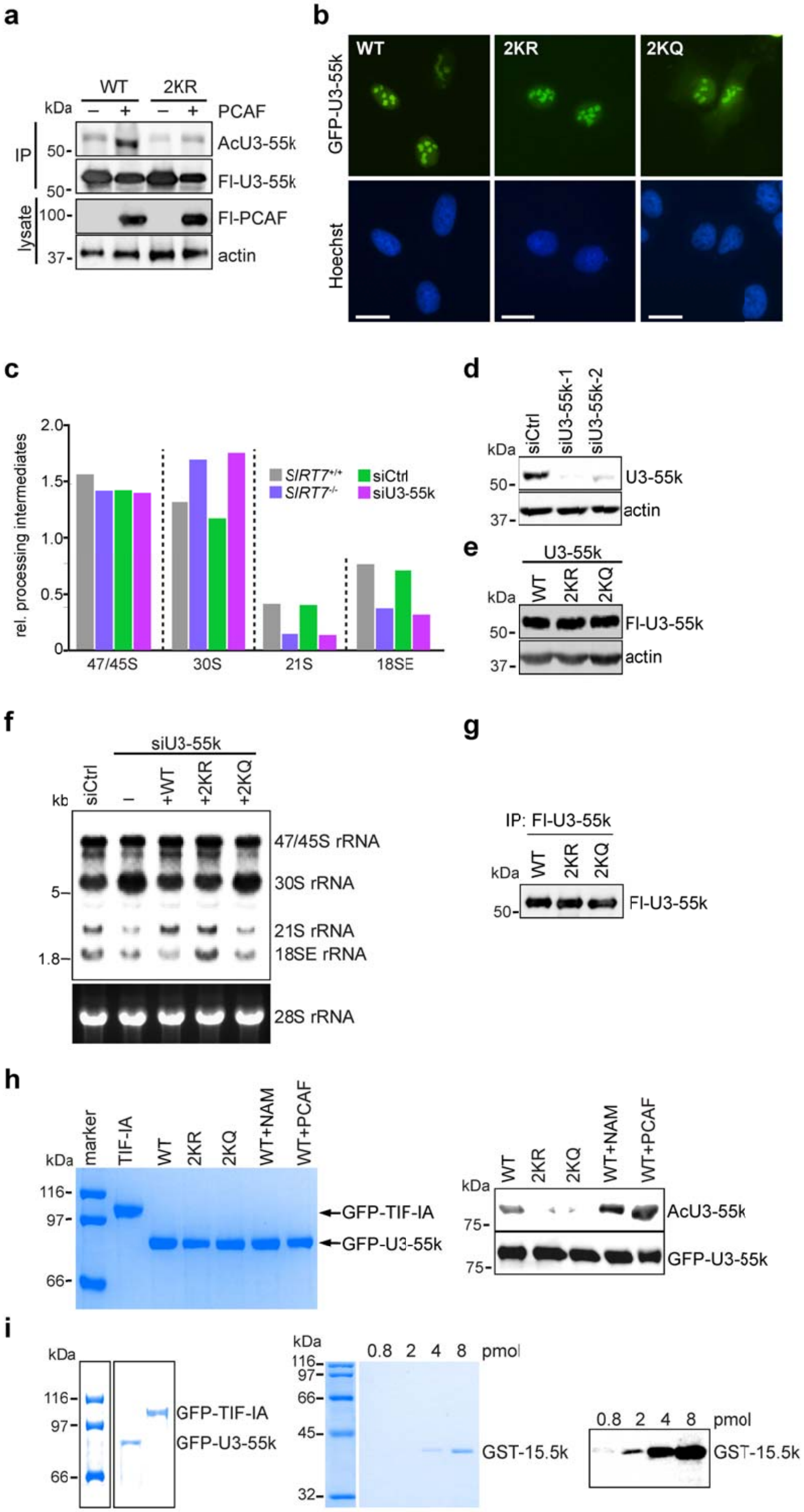
a
WT
2KR
kDa
PCAF
IP
lysate
AcU3-55k
Fl-U3-55k
Fl-PCAF
actin
b
WT
2KR
2KQ
GFP-U3-55k
Hoechst
c
rel. processing intermediates
SIRT7+/+
SIRT7-/-
siCtrl
siU3-55k
47/45S
30S
21S
18SE
d
siCtrl
siU3-55k-1
siU3-55k-2
U3-55k
actin
e
U3-55k
Fl-U3-55k
actin
f
siU3-55k
siCtrl
+WT
+2KR
+2KQ
kb
47/45S rRNA
30S rRNA
21S rRNA
18SE rRNA
28S rRNA
g
IP: Fl-U3-55k
Fl-U3-55k
h
marker
TIF-IA
WT+NAM
WT+PCAF
GFP-TIF-IA
GFP-U3-55k
AcU3-55k
i
GFP-TIF-IA
GFP-U3-55k
pmol
GST-15.5k

Supplementary Figure 5. **SIRT7-mediated deacetylation of U3-55k promotes U3 snoRNA binding.**

(**a**) PCAF acetylates U3-55k at lysine 12 and 25. Flag-tagged wildtype (WT) or mutant U3-55k (2KR) were expressed in HEK293T cells either alone or together with Flag-PCAF, immunoprecipitated and subjected to western blot analysis with anti-pan-AcK and anti-Flag antibodies (IP panels). Lysates were probed with anti-Flag to detect expression of Flag-PCAF, and reprobed with anti-actin to confirm equal loading (lower panels). (**b**) Cellular localization of wildtype and mutant U3-55k. U2OS cells transiently expressing GFP-tagged wildtype (WT) or mutant U3-55k (2KR or 2KQ) were imaged for GFP-signal distribution (*n*=120). Nuclei were stained with Hoechst 33342. Scale bars, 10 µm. (**c**) Quantitation of the northern blot signals presented in **Fig. 4c** by PhosphorImaging. (**d**) Western blot demonstrating knockdown of endogenous U3-55k by two different siRNAs (see **Supplementary Table 2**). (**e**) Western blot confirming similar expression levels of wildtype (WT) and mutant (2KR and 2KQ) GFP-U3-55k. Related to **Fig. 4d**. (**f**) Northern blot of RNA in cells depleted of U3-55k. HEK293T cells depleted of U3-55k by U3-55k siRNAs against the 3'-UTR were transfected with Flag-tagged wildtype U3-55k, U3-55k/2KR or 2KQ mutant. RNA was hybridized to ^{32}P-labeled ITS1 oligo to monitor processing intermediates. (**g**) Western blot of immunoprecipitated Flag-tagged wildtype (WT) and mutant (2KR and 2KQ) U3-55k used in the CLIP assays in **Fig. 4e**. (**h**) Coomassie-stained PAA gel of purified bead-bound GFP-TIF-IA (TIF-IA), wildtype (WT) and mutant (2KR, 2KQ) GFP-tagged U3-55k used in the RNA pull-down assays presented in **Fig. 4f**. As indicated, GFP-U3-55k/WT was purified from cells treated with NAM for 6 h (WT+NAM) or from cells co-expressing Flag-PCAF (WT+PCAF). Broad range protein standards were used as marker. In the right panel, the acetylation level of wildtype GFP-U3-55k or the 2KR and 2KQ mutants was detected in western blots with anti-pan-AcK antibody (top). Equal loading of GFP-tagged U3-55k proteins was confirmed with anti-GFP antibody (bottom). (**i**) GFP-U3-55k, GFP-TIF-IA, and GST-15.5k was analyzed by coomassie staining (left, middle) and western blotting using anti-GST antibody (right). Related to **Fig. 4h**.

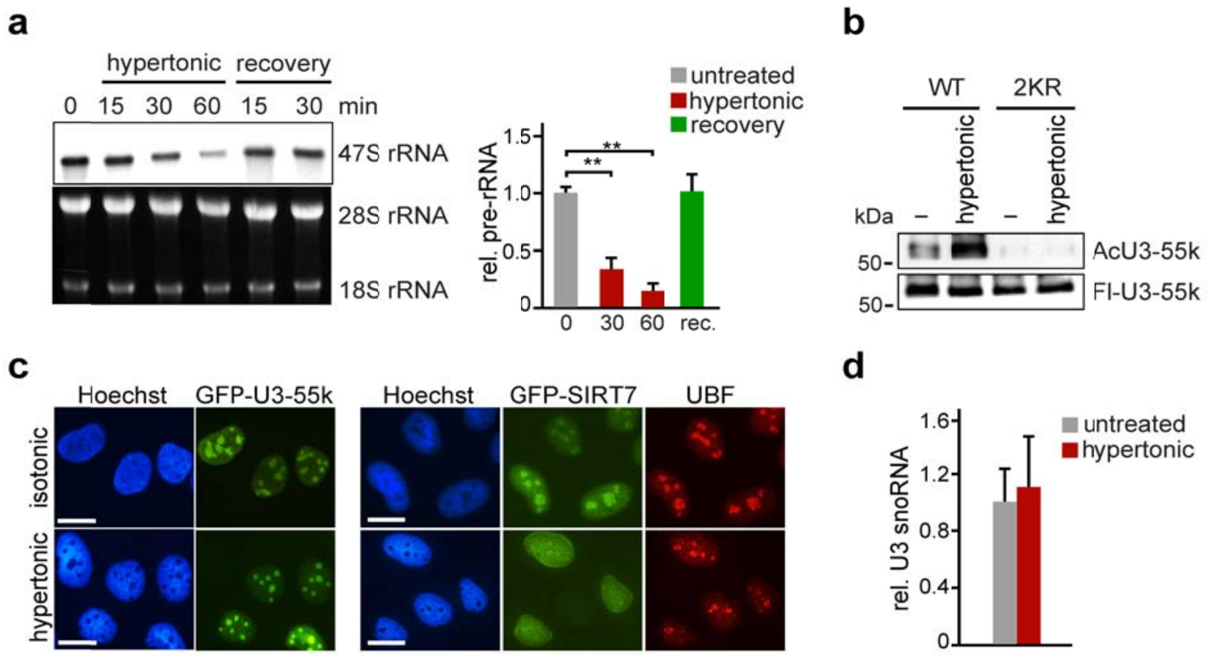

Supplementary Figure 6. Stress-induced reduction in pre-rRNA synthesis and processing correlates with acetylation of U3-55k.

(**a**) Northern blot of pre-rRNA detected by a 5'ETS-specific riboprobe from HEK293T cells that were unperturbed or subjected to hypertonic stress for the indicated time. Restoration of isotonicity for 15 or 30 minutes, after 60 minutes of stress, led to recovery of pre-rRNA levels. The bottom panel shows an ethidium bromide stain confirming equal loading of RNA. Pre-rRNA levels were further confirmed by RT-qPCR normalized to actin mRNA (right panel). Bars represent means ±SD from three biological repeats (***P*<0.01). (**b**) Lysines 12 and 25 of U3-55k are hyperacetylated upon stress. HEK293T cells expressing wildtype (WT) or mutant (2KR) U3-55k were exposed to hypertonic stress. Acetylation and the amount of immunopurified U3-55k were analyzed on western blots using anti-pan-AcK and anti-Flag antibodies. (**c**) Cellular localization of U3-55k is not affected by hypertonic stress. Fluorescence images of GFP-U3-55k (left) or GFP-SIRT7 (right) in U2OS cells before and after exposure to hypertonic stress for 1 h. Nuclei were stained with Hoechst 33342. UBF was visualized by immunofluorescence using antibodies specific to UBF (right). Scale bars, 10 µm. (**d**) RT-qPCR analysis of U3 snoRNA level under hypertonic stress. The amount of snoRNA was normalized to actin mRNA. Bars represent means ±SD from three biological repeats. See also **Fig. 5e**.

Uncropped blots related to **Fig. 3a**

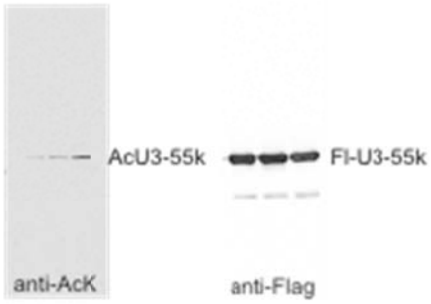

Uncropped blots related to **Fig. 3b**

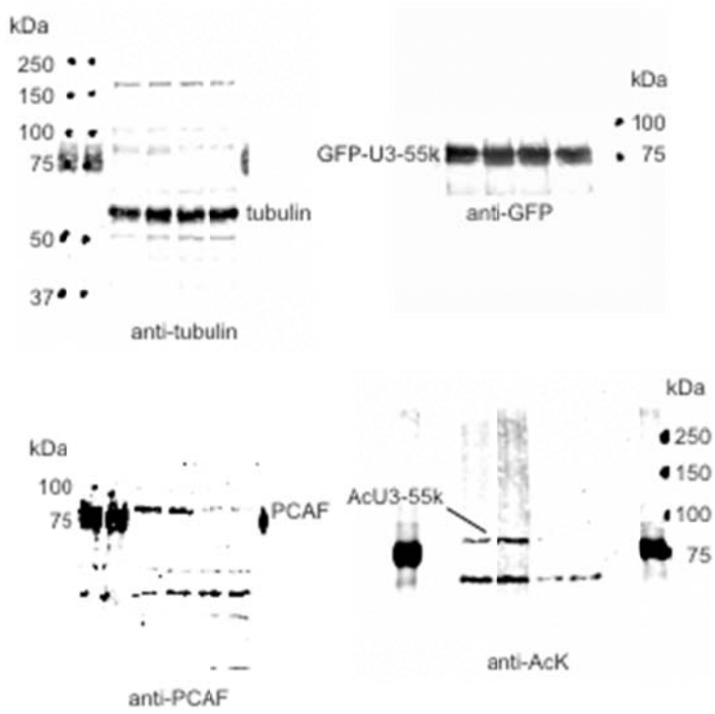

Uncropped blots related to **Fig. 3c**

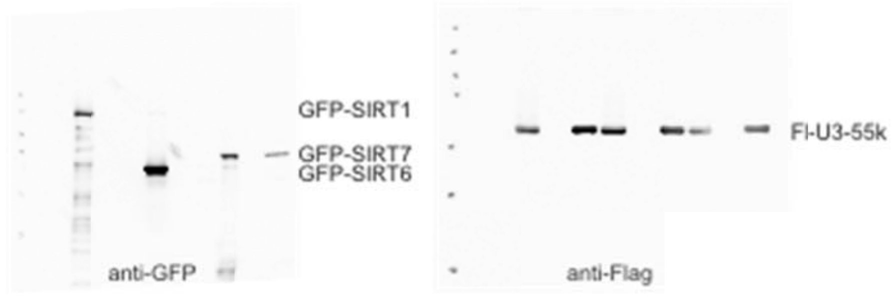

Uncropped blots related to **Fig. 3d**

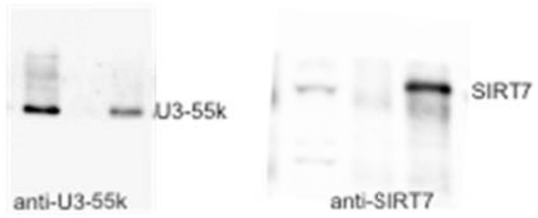

Uncropped blots related to **Fig. 3e**

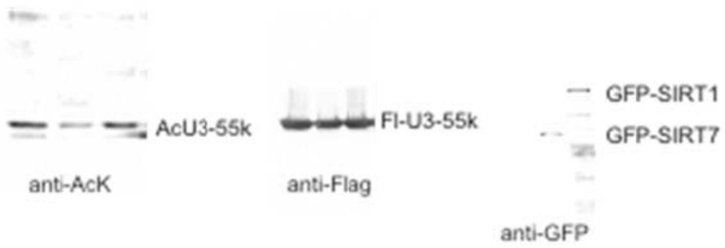

Uncropped blots related to **Fig. 3f** (left panel)

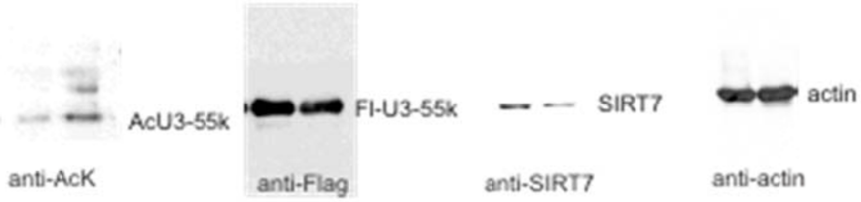

Uncropped blots related to **Fig. 3f** (right panel)

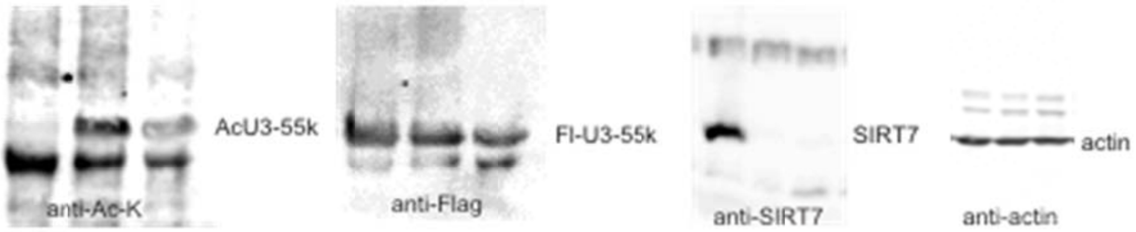

Uncropped blots related to **Fig. 3g**

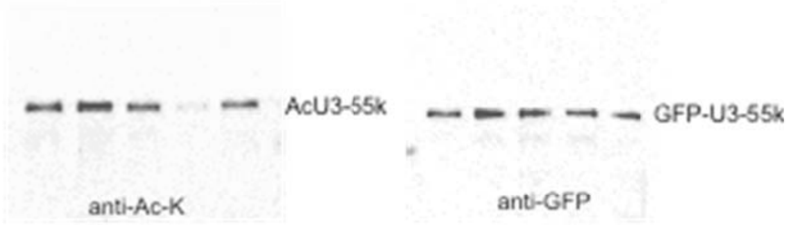

Uncropped blots related to **Fig. 4b**

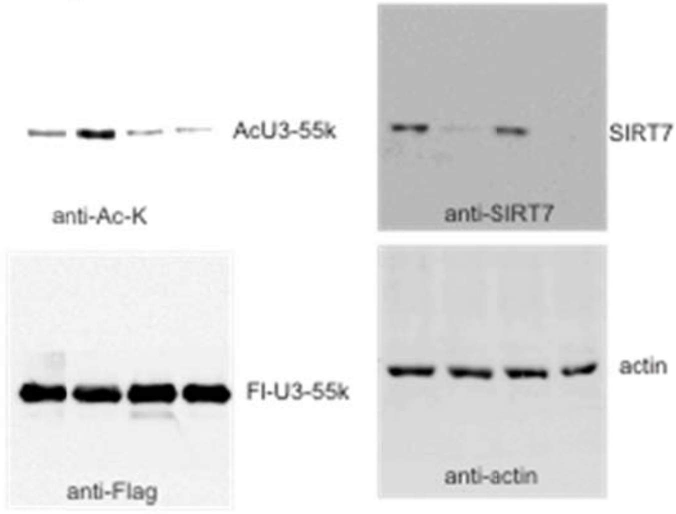

Uncropped gel related to **Fig. 4f**

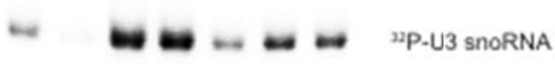

Uncropped gel and blot related to **Fig. 4g**

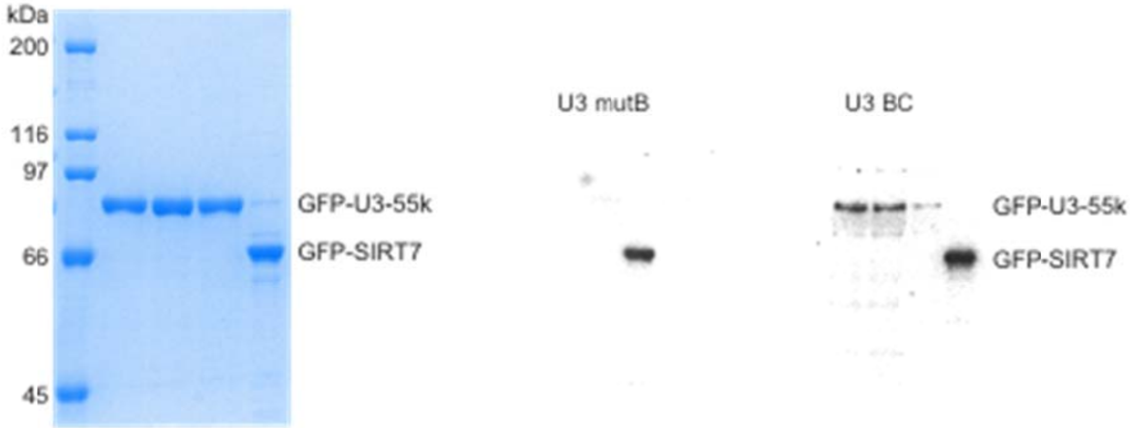

Uncropped gels related to **Fig. 4h**

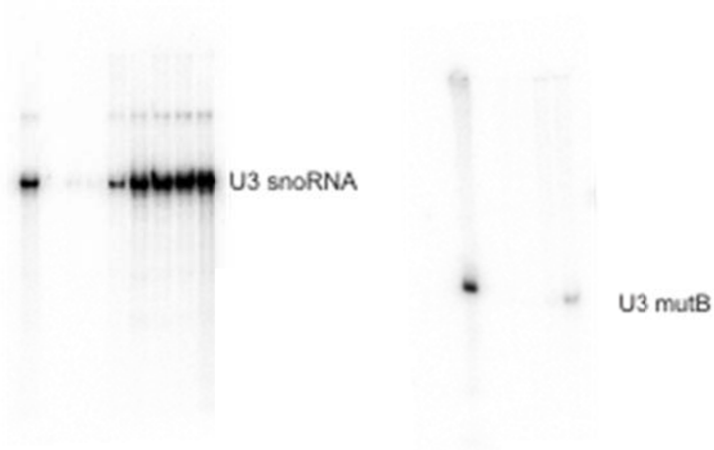

Uncropped blots and gel related to **Fig. 5a**

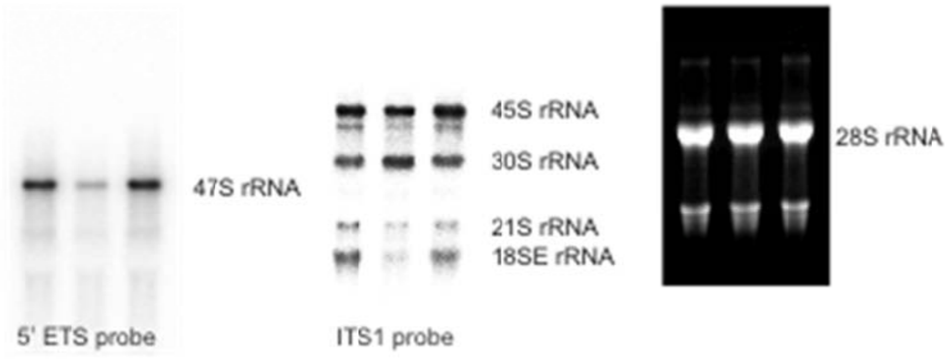

Uncropped blots related to **Fig. 5b**

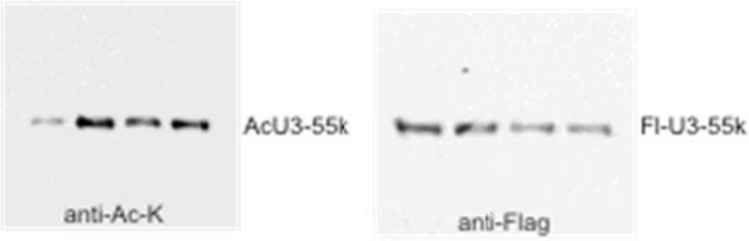

Uncropped blots related to **Fig. 5d**

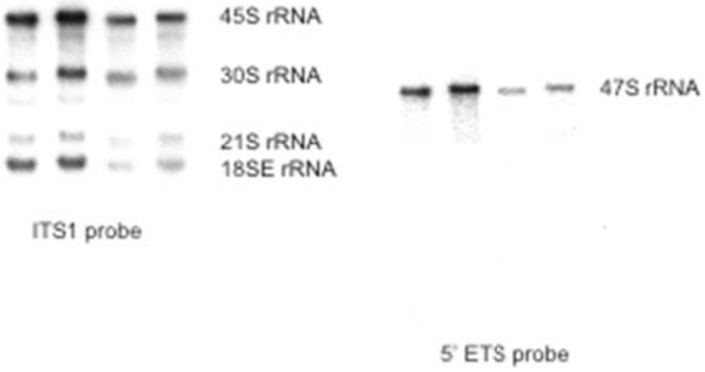

Uncropped blots and gel related to **Fig. 5g**

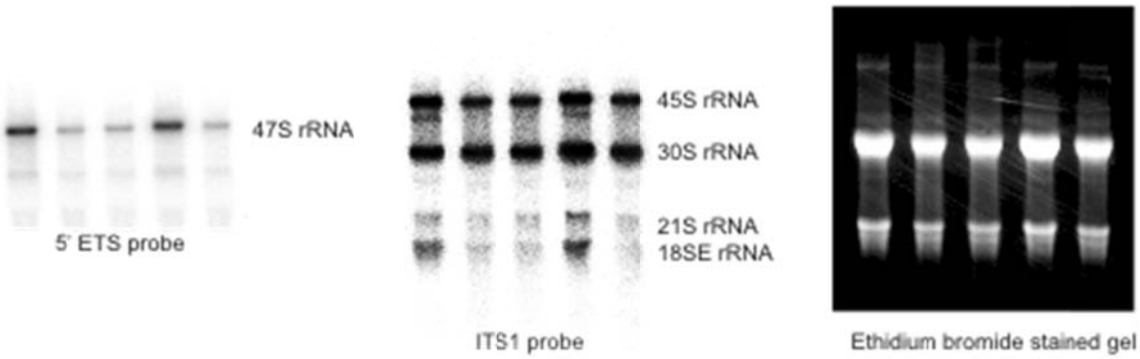

Supplementary Figure 7. Uncropped blots and gels related to Fig. 3, 4 and 5.

Supplementary Table 1. List of snoRNAs identified in the SIRT7 CLIP reads.

Chrom	ChromStart	ChromEnd	Strand	Exon size (bp)	Name	Alternative name	Host gene	snoRNA type
12	6619387	6619717	+	330	SCARNA10	U85	NCAPD2	sca with C/D and H/ACA domains
14	95999691	95999966	-	275	SCARNA13	U93	SNHG10	sca
4	1976362	1976487	+	125	SCARNA23	ACA12	POLA	sca H/ACA box
2	234184372	234184648	+	276	SCARNA5	U87	APG16L	sca with C/D and H/ACA domains
2	234197321	234197586	+	265	SCARNA6_1	U88	APG16L	sca with C/D and H/ACA domains
16	2012334	2012467	-	133	SNORA10	ACA10	RPS2	H/ACA
X	51933716	51933844	+	128	SNORA11D	SNORA11D	MAGED4	H/ACA
1	28907431	28907566	-	135	SNORA16A	ACA16	SNHG12	H/ACA
7	6056507	6056642	+	135	SNORA42_4	ACA42	AIMP2	H/ACA
12	98993412	98993661	+	249	SNORA53	ACA53	SLC25A3	H/ACA
11	2985000	2985123	-	123	SNORA54	ACA54	NAP1L4	H/ACA
7	45143947	45144081	-	134	SNORA5A	ACA5	TBRG4	H/ACA
7	45144504	45144641	-	137	SNORA5C	ACA5c	TBRG4	H/ACA
1	28833877	28834083	+	206	SNORA73A	U17a	RCC1	H/ACA
1	28835070	28835274	+	204	SNORA73B	U17b	RCC1	H/ACA
5	138614469	138614667	+	198	SNORA74A	U19	MATR3	H/ACA
22	34100771	34100906	-	135	SNORA76_2	ACA62	ESTs cluster	H/ACA
22	20113924	20114049	+	125	SNORA77_4	ACA63	RANBP1	H/ACA
16	2015184	2015311	+	127	SNORA78	ACA64	SNHG9	H/ACA
14	81669038	81669178	-	140	SNORA79_1	ACA65	GTF2A1	H/ACA
21	33749495	33749631	-	136	SNORA80	ACA67	URB1	H/ACA
17	62223442	62223512	+	70	SNORD104	U104	spliced ESTs cluster	C/D
20	47895476	47895565	+	89	SNORD12C	U106	ZFAS1	C/D
8	33370992	33371096	+	104	SNORD13	U13	-	C/D
11	122930042	122930130	-	88	SNORD14C	U14C	HSPA8	C/D
11	122929616	122929703	-	87	SNORD14D	U14D	HSPA8	C/D
20	17943352	17943589	-	237	SNORD17	HBI-43	SNX5	C/D
19	48259109	48259219	+	110	SNORD23_1	HBII-115	GLTSCR2	C/D
19	49993871	49993956	+	85	SNORD33_1	U33	RPL13A	C/D
19	49994160	49994231	+	71	SNORD34	U34	RPL13A	C/D

19	49994431	49994517	+	86	SNORD35A	U35A	RPL13A	C/D
9	136217701	136217767	+	66	SNORD36C	U36C	PRL7A	C/D
1	45243514	45243584	+	70	SNORD38A	U38A	RPS8	C/D
17	19091328	19092027	+	699	SNORD3A	U3	-	C/D
17	18965224	18965807	+	583	SNORD3B-1	U3	-	C/D
12	57038810	57038885	-	75	SNORD59A	U59A	ATP5B	C/D
16	2205023	2205106	-	83	SNORD60_2	U60	ESTs cluster	C/D
9	134361051	134361137	+	86	SNORD62A	U62A	PRRC2B	C/D
9	134365872	134365958	+	86	SNORD62B	U62B	PRRC2B	C/D
2	203141153	203141241	+	88	SNORD70	HBII-234	NOP58	C/D
14	21865450	21865560	-	110	SNORD8	mgU6-53	CHD8	C/D
20	2636742	2636828	+	86	SNORD86	HBII-202	NOP56	C/D
19	51302288	51302379	-	91	SNORD88B	HBII-180B	C19orf48	C/D

Supplementary Table 2. Sequences of siRNAs used in this study.

Name	RNA sequences (5' to 3')
U3-55k siRNA-1 (3'UTR)	sense: CCACCCUCUUUGUAUUAAA<br>antisense: UUUAAUACAAAGAGGGUGG
U3-55k siRNA-2 (3'UTR)	sense: UUUAAGUCCUUCCCAGGCU<br>antisense: AGCCUGGGAAGGACUUAAA

Supplementary Table 3. Oligos used in this study.

Oligo name	Sequence (5' to 3')	Purpose
ß-actin mRNA F[1]	CGTCACCAACTGGGACGACA	qPCR
ß-actin mRNA R[1]	CTTCTCGCGGTTGGCCTTGG	
U2 snRNA F[2]	TTTGGCTAAGATCAAGTGTAGTATCTGTTC	qPCR
U2 snRNA R[2]	AATCCATTTAATATATTGTCCTCGGATAGA	
U3 snoRNA F[3]	AGAGGTAGCGTTTTCTCCTGAGCG	qPCR
U3 snoRNA R[3]	ACCACTCAGACCGCGTTCTC	
U13 snoRNA F[4]	CTTTTGTAGTTCATGAGCGTG	qPCR
U13 snoRNA R[4]	GGTCAGACGGGTAATGTGC	
U14 snoRNA F[5]	TGGTTTTCCAACATTCGCAG	qPCR
U14 snoRNA R[5]	ATCCAAGGAAGGTTTACCCA	
SCARNA6 F	GTCCCTCATTCTGTGTTCCTC	qPCR
SCARNA6 R	GCAGATCATAGCCACAGATACC	
SCARNA10 F	TCTTGGTGGGCGATACAGAGTTA	qPCR
SCARNA10 R	CTTGGCCCTGATACCCTGAACAT	
SCARNA13 F	CCTCTGATAGTTTCTGGTCACTG	qPCR
SCARNA13 R	AGCTTCTCTTACTGTTGGCG	
SNORA73A (U17A) F	AATAAAGCTGGGCCTCGTGTCTG	qPCR
SNORA73A (U17A) R	GCTGTTTCCTGCATGGTTTGTCTC	
SNORA74A F	CACTGTCTTTATTGAGGTTTGGC	qPCR
SNORA74A R	GTCATTCCACTCTCAGAGGTG	
SNORD88B F	GACCCCGTGATGTCCAG	qPCR
SNORD88B R	AGAACCCCGGATGTCAAAG	
pre-U3 snoRNA R (a)	AAGGAAAAACCACTCAGA	qPCR
pre-U3 snoRNA R (b)	AAGGAAAAACCACTCA	qPCR
pre-rRNA F[1]	TGTCAGGCGTTCTCGTCTC	qPCR
pre-rRNA R[1]	AGCACGACGTCACCACATC	

rDNA H1 F[6]	GGCGGTTTGAGTGAGACGAGA	qPCR
rDNA H1 R[6]	ACGTGCGCTCACCGAGAGCAG	
rDNA H4 F[6]	CGACGACCCATTCGAACGTCT	qPCR
rDNA H4 R[6]	CTCTCCGGAATCGAACCCTGA	
rDNA H18 F[6]	GTTGACGTACAGGGTGGACTG	qPCR
rDNA H18 R[6]	GGAAGTTGTCTTCACGCCTGA	
rDNA +541/T7 F (mouse)	TAATACGACTCACTATAGGGAGGTCGCTCGTTGTGTTCTC TTG	cloning
rDNA +1290 R (mouse)	AAACTTTCCAACCCCAGCCGCG	cloning
rDNA +709/T7 F (mouse)	TAATACGACTCACTATAGGGAGGGCGAGGGACGGACATT CA	cloning
rDNA +1060 R (mouse)	AGGAGACAAACCTGGAACG	cloning
Flag-U3-55k F	CGCGGATCCCATGTCGGCAACAGCGGCTGCTCGTA	cloning
Flag-U3-55k R	CACAGGGGCCCTCAGGAACCAGCAGCTGGGGGTACA	
GFP- U3-55k F	CACAGGGGCCCATGTCGGCAACAGCGGCTGCTCGTA	cloning
GFP- U3-55k R	CGCGGATCCTCAGGAACCAGCAGCTGGGGGTACA	
U3-55k/K12R F	GCGGGGACGGCCGGCCTCTGGGGCC	mutagenesis
U3-55k/K12R R	AGGCCGGCCGTCCCCGCTTACGAGC	
U3-55k/K12Q F	GCGGGGACAGCCGGCCTCTGGGGCC	mutagenesis
U3-55k/K12Q R	AGGCCGGCTGTCCCCGCTTACGAGC	
U3-55k/K25R F	GGGGGCCGGCCGGCGGCGGCGAAAG	mutagenesis
U3-55k/K25R R	CTTTCGCCGCCGCCGGCCGGCCCCC	
U3-55k/K25Q F	GGGGGCCGGCCAGCGGCGGCGAAAG	mutagenesis
U3-55k/K25Q R	CTTTCGCCGCCGCTGGCCGGCCCCC	
sgSIRT7-(1) F[7]	CACCGTGGTAACGGAGCTGCAGGGC	CRISPR/Cas9
sgSIRT7-(1) R[7]	AAACGCCCTGCAGCTCCGTTACCA	
sgSIRT7-(2) F[8]	CACCGCGAGCGCAAAGCGGCGGAGC	CRISPR/Cas9
sgSIRT7-(2) R[8]	AAACGCTCCGCCGCTTTGCGCTCG	
sgSIRT7-(3) F[8]	CACCGGCGGGTCCGGAGGTTGCGGG	CRISPR/Cas9
sgSIRT7-(3) R[8]	AAACCCCGCAACCTCCGGACCCGC	
sgSIRT7-(4) F[8]	CACCGGCGCATCCTGAGGAAGGCGG	CRISPR/Cas9
sgSIRT7-(4) R[8]	AAACCCGCCTTCCTCAGGATGCGC	
mSIRT7 shRNA-1 sense (mouse)	CCGGAAACTCTACATCGTGAACCTGCTCGAGCAG GTTCACGATGTAGAGTTTTTTTG	cloning of shRNA
mSIRT7 shRNA-1 antisense (mouse)	AATTCAAAAAAACTCTACATCGTGAACCTGCTCGAGCAG GTTCACGATGTAGAGTTT	
mSIRT7 shRNA-2 sense (mouse)	CCGGCCTCCCTCTTTCTACTCCTTACTCGAGTAAGGAGTA GAAAGAGGGAGGTTTTTG	cloning of shRNA
mSIRT7 shRNA-2 antisense (mouse)	AATTCAAAAACCTCCCTCTTTCTACTCCTTACTCGAGTAAG GAGTAGAAAGAGGGAGG	

shRNA scrambled sense (mouse)	CCGGCAACGAAGGAATTCTTCGGCCCTCGAGGGCCGAAGAATTCCTTCGTTGTTTTTG	cloning of shRNA
shRNA scrambled antisense (mouse)	AATTCAAAAACAACGAAGGAATTCTTCGGCCCTCGAGGGCCGAAGAATTCCTTCGTTG	
U2 snRNA ASO (mouse)[9]	CAGATACTACACTTG	depletion
U3 snoRNA ASO (mouse)[9]	GTGGTTTCGGGTGCTC	depletion
ITS1	CCTCGCCCTCCGGGCTCCGTTAATGATC	hybridization

Unless otherwise indicated all oligos correspond to human sequences.

Supplementary References

1. Chen, S. *et al.* Repression of RNA polymerase I upon stress is caused by inhibition of RNA-dependent deacetylation of PAF53 by SIRT7. *Mol. Cell* **52**, 303-313 (2013).

2. Zhang, Z. *et al.* SMN deficiency causes tissue-specific perturbations in the repertoire of snRNAs and widespread defects in splicing. *Cell* **133**, 585-600 (2008).

3. Scott, M.S. *et al*. Human box C/D snoRNA processing conservation across multiple cell types. *Nucleic Acids Res.* **40**, 3676-3688 (2012).

4. Nallar, S.C. *et al*. GRIM-1, a novel growth suppressor, inhibits rRNA maturation by suppressing small nucleolar RNAs. *PLoS ONE* **6**, e24082 (2011).

5. Padilla, P.I. *et al*. Association of guanine nucleotide-exchange protein BIG1 in HepG2 cell nuclei with nucleolin, U3 snoRNA, and fibrillarin. *Proc. Natl. Acad. Sci. USA* **105**, 3357-3361 (2008).

6. O'Sullivan, A.C., Sullivan, G.J. & McStay, B. UBF binding in vivo is not restricted to regulatory sequences within the vertebrate ribosomal DNA repeat. *Mol. Cell. Biol.* **22**, 657-668 (2002).

7. Shalem, O. *et al.* Genome-scale CRISPR-Cas9 knockout screening in human cells. *Science* **343**, 84-87 (2014).

8. Wang, T., Wei, J.J., Sabatini, D.M. & Lander, E.S. Genetic screens in human cells using the CRISPR-Cas9 system. *Science* **343**, 80-84 (2014).

9. Kass, S., Tyc, K., Steitz, J.A. & Sollner-Webb, B. The U3 small nucleolar ribonucleoprotein functions in the first step of pre-ribosomal RNA processing. *Cell* **60**, 897-908 (1990).
